# Thermoelectric transport in Weyl semimetals under a uniform concentration of torsional dislocations†

**DOI:** 10.1039/d4na00056k

**Published:** 2024-04-03

**Authors:** Daniel A. Bonilla, Enrique Muñoz

**Affiliations:** a Facultad de Física, Pontificia Universidad Católica de Chile Vicuña Mackenna 4860 Santiago Chile ejmunozt@uc.cl; b Center for Nanotechnology and Advanced Materials CIEN-UC Avenida Vicuña Mackenna 4860 Santiago Chile

## Abstract

In this article, we present an effective continuum model for a Weyl semimetal, to calculate its thermal and thermoelectric transport coefficients in the presence of a uniform concentration of torsional dislocations. We model each dislocation as a cylindrical region of finite radius *a*, where the corresponding elastic strain is described as a gauge field leading to a local pseudo-magnetic field. The transport coefficients are obtained by a combination of scattering theory, Green's functions and the Kubo formulae in the linear response regime. We applied our theoretical results to predict the electrical and thermal conductivities as well as the Seebeck coefficient for several transition metal monopnictides, *i.e.* TaAs, TaP, NbAs and NbP.

## Abstract 2

Thermoelectric transport coefficients in transition metal monopnictides with a uniform concentration of torsional dislocations.

## Graphical abstract

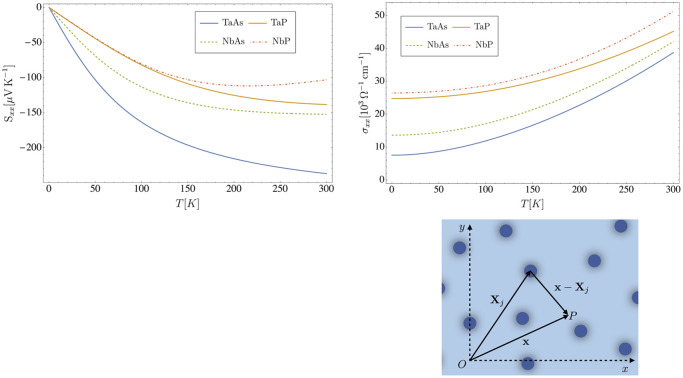

## 1 Introduction

Not long after being postulated as a theoretical concept,^1–7^ Weyl semimetals (WSMs) were discovered in TaAs crystals.^8^ WSMs constitute important examples of three-dimensional, gapless materials with non-trivial topological properties, as their band structure displays an even number of Weyl nodes. Near each node, the charge carriers are massless quasi-particles with linear dispersion and pseudo-relativistic properties.^4–7^ In particular, each node is a monopolar source of Berry curvature, and hence they are protected from being gaped since their topological charge (chirality) is an invariant.^7^ This implies that in Weyl fermions, the projection of spin over their momentum direction is preserved, a condition referred to as “spin-momentum locking”.

Some remarkable properties related to the existence of Weyl nodes in the bulk band structure are the presence of Fermi arcs,^8^ the chiral anomaly, and the chiral magnetic effect.^9^ As a consequence, in recent years considerable effort has been devoted to the study of the electronic transport properties of WSMs, including the effects of different scattering mechanisms, such as electron–phonon and localized impurities.^10–16^ Different estimations in the literature report first-principles calculations for the optical conductivities in the monopnictide family (TaAs, TaP, NbAs and NbP),^17–22^ which in the low-frequency (DC) limit are in the range *σ*_*xx*_ ∼ 10^4^ to 10^6^ Ω^−1^ cm^−1^ (see Table 1). Concerning the electronic contribution to the thermal conductivity, including the aforementioned scattering mechanisms, estimations based on first-principles calculations^23–25^ report values in the range *κ*^(el)^_*xx*_ ∼ 20–100 W K^−1^ m^−1^. The lattice contribution, on the other hand, is strongly dependent on the masses of the nuclei, and hence it varies in a wider range for the different materials *κ*^(*l*)^_*xx*_ ∼ 1–190 W K^−1^ m^−1^ (see Table 1). It has been proposed that generic semi-metals may constitute attractive candidates for thermoelectric applications due to their relatively large Seebeck coefficients at room temperature |*S*| ∼ 10^2^ μV K^−1^.^26^ This parameter is very sensitive to the density of carriers through the chemical potential, but different estimations in the literature for the family of transition metal monopnictides report values in the range |*S*| ∼ 10^2^ to 10^3^ μV K^−1^ (ref. 23–25) at room temperature. Therefore, the general concept of “Topological Thermoelectrics” has generated a lot of interest in the materials science community, with excellent recent reviews^27^ on the subject.

**Table 1:** Values of the DC conductivity *σ*_*xx*_, the electronic *κ*^(el)^_*xx*_, and lattice *κ*^(*l*)^_*xx*_ contributions to the thermal conductivities at 300 K reported in the literature

Material	*σ*_*xx*_ (10^4^ Ω^−1^ cm^−1^)	*κ* ^(el)^_*xx*_ (W K^−1^ m^−1^)	*κ* ^(*l*)^_*xx*_ (W K^−1^ m^−1^)
TaAs	∼1–10 (ref. 19)	56.87 (ref. 23)	36.06 (ref. 23)
TaP	12.5 (ref. 20)	∼100 (ref. 24)	∼190 (ref. 24)
NbAs	307.6 (ref. 21)	21.2 (ref. 25)	1.37 (ref. 23)
NbP	102 (ref. 22)	33.8 (ref. 25)	1.99 (ref. 25)

In contrast with results reported in the literature, the effects of mechanical strain and dislocations or disclinations have been theoretically explored to a much lesser extent in the context of electronic and thermal transport properties. Those defects can be modeled in a continuum approximation by gauge fields^28–30^ in WSMs. More recently, the role of gauge fields has been explored in acoustic crystal realizations of topological materials as well,^31^ particularly in their role in representing topological defects.^32^

In our previous studies, we have studied quasi-ballistic transport through a nano-junction in a WSM with a single torsional dislocation, in combination with an external magnetic field. For such a system, we obtained the electronic^33,34^ and thermoelectric^33,35^ transport coefficients, using the Landauer ballistic formalism in combination with a mathematical analysis for the quantum mechanical scattering cross-section.^36^ More recently, we considered the case of a diluted, uniform concentration of torsional dislocations and their effects on the electrical conductivity of type I WSMs,^37^ by means of the Kubo linear-response formalism. The effect of the random distribution of dislocations, with a concentration *n*_d_ (per unit area), is incorporated in the form of a disorder-averaged self-energy into the corresponding Dyson's equation for the retarded and advanced Green's functions. Furthermore, as described in ref. 37 a vertex correction obtained as a solution to the Bethe–Salpeter equation was incorporated into the Kubo linear response formulae.

In the present work, our purpose is to further extend this study, in combination with Onsager relations of non-equilibrium thermodynamics, to obtain the electronic component of the thermal conductivity and Seebeck coefficient in these materials, limited by this particular scattering mechanism, as a function of temperature and concentration of dislocations. We remark that this is the single scattering mechanism that we shall focus on this study, since it requires a special modeling as compared to other mechanisms that have been already discussed extensively in the literature. Moreover, as we state in the Discussion, Mathiessen's rule allows one to combine all these different contributions *via* the overall relaxation time in the estimation of the transport coefficients.

We present explicit evaluations of our analytical expressions for the electrical and thermal conductivity, as well as for the Seebeck coefficient, as a function of temperature and concentration of dislocations *n*_d_, for several materials in the family of transition metal monopnictides, *i.e.* TaAs, TaP, NbAs and NbP, with microscopic parameters estimated from *ab initio* calculations as reported in the literature.^17,38,39^ Our calculations show that, although the Wiedemann–Franz law is satisfied for all such compounds in the low-temperature limit, the Seebeck coefficient leads to a large figure of merit *ZT*^(el)^ > 2 even at room temperature for TaAs. Therefore, our theoretical results suggest that the transition metal monopnictides may constitute very attractive candidates for thermoelectric applications in energy harvesting. Since our model only captures the electronic contribution to the thermal conductivity, this possibility must be further explored to evaluate in more detail the role of phonon-related scattering effects and lattice thermal conductivity.

## 2 Scattering by a uniform concentration of dislocations

**Fig. 1:**
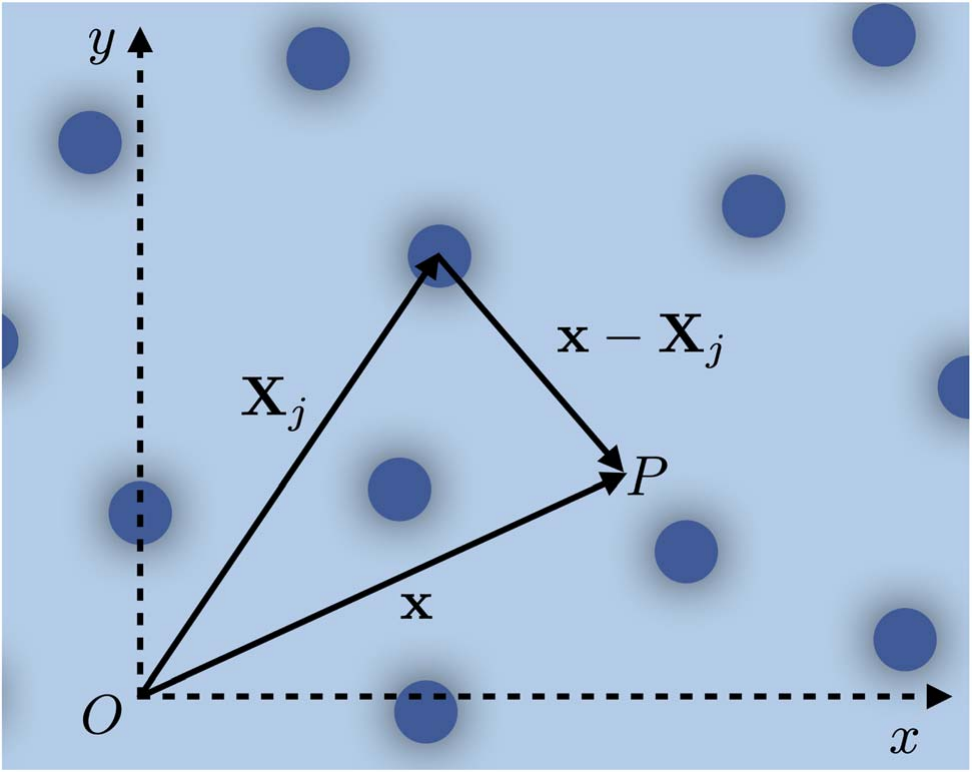
Random distribution of torsional dislocations, as seen from a plane perpendicular to the cylinder axis. Each dislocation is modeled as a cylinder of radius *a*, whose central axis is located at the vector **X**_*j*_ (on the perpendicular plane *x*–*y*). The position of an arbitrary point *P* on the plane is **x** = (*x*, *y*), and we define *r*_*j*_ = |**x** − **X**_*j*_| as its relative distance from the *j*^th^ dislocation axis.

Let us start with an effective continuum model for a type I WSM, in the presence of a uniform concentration *n*_d_ = *N*_d_/*A* (per unit transverse surface) of identical cylindrical dislocations of finite radius *a*, as depicted in Fig. 1. The spatial distribution of such defects is represented by the density function1
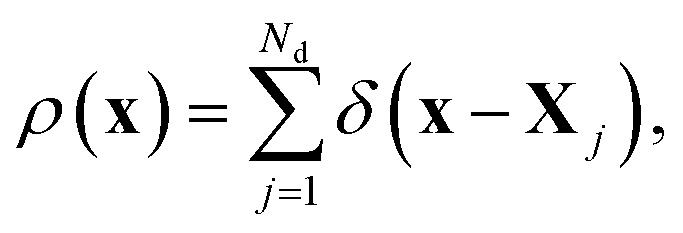
where **X**_*j*_ is the position of the *j*^th^-dislocation's axis. We model this system using the Hamiltonian^37^2
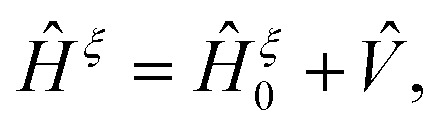
where3
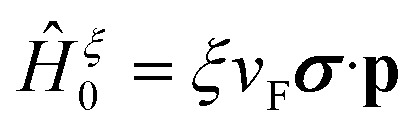


for ***σ*** = (*σ*_*x*_, *σ*_*y*_, *σ*_*z*_) the vector of Pauli matrices represents the free-particle Hamiltonian at each of the Weyl nodes **K**_±_ = ±**b**/2, labeled by their corresponding chirality index *ξ* = ±, and *v*_F_ is the Fermi velocity. In addition, *V̂* represents the scattering potential due to the presence of the random distribution of dislocations,4
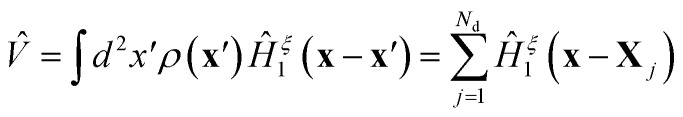


given that the contribution from a single dislocation defect is given by^34–37^5
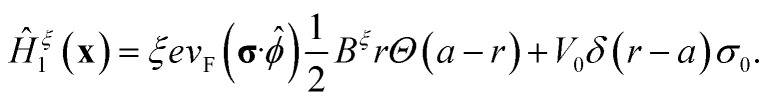
Here, *r*_*j*_ = |**x** − **X**_*j*_| is the distance from the center of each dislocation 1 ≤ *j* ≤ *N*_d_ (see Fig. 1), and *φ̂* = (−sin *ϕ*,cos *ϕ*,0) the azimuthal unit vector in polar coordinates. Eqn (5) contains the interaction with each cylindrical dislocation of radius *a*, where torsional strain is described as a pseudo-magnetic field **B**^*ξ*^ in its interior *r* < *a*,^34–37^ as described by the Heaviside function *Θ*(*a* − *r*). The corresponding lattice mismatch effect at the boundary *r* = *a* is represented by a repulsive delta barrier^35,37^ with strength *V*_0_. In this formalism, the pseudo-magnetic field **B**^*ξ*^ = ∇ × **A**^*ξ*^ representing strain is proportional to the torsional angle *θ* (in degrees), a relation that is convenient to express in terms of its flux through the circular cross-section of each cylindrical region: |**B**^*ξ*^|*a*^2^ = 1.36*θφ̃*_0_.^35^ Here, we defined the modified flux quantum representing the dislocations in these materials by 
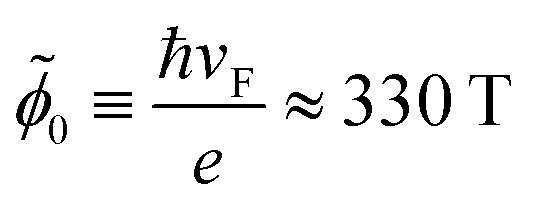
 Å^2^.^35^

As described in detail in ref. 37, we include the effect of disorder by taking the *configurational average* over the statistical distribution of dislocations, defined as6
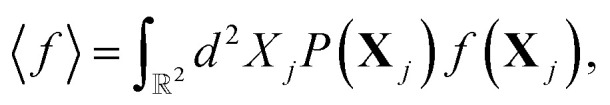
where *f*(**X**_*j*_) is any function of the dislocations' positions and *P*(**X**_*j*_) is their statistical distribution function in the sample. In particular, for a uniform distribution we have *P*(**X**_*j*_) = 1/*A*, where *A* is the area of the plane normal to each cylinder's axis.

As we shall present in the next section, for the calculation of the thermal and thermoelectric transport coefficients in this material, we are interested in the disorder-averaged retarded Green's function7
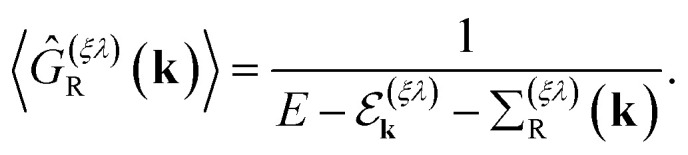
Here 
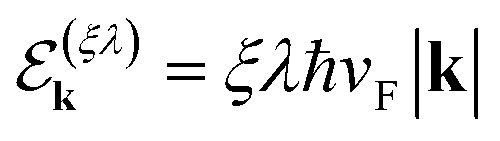
 is the energy spectrum of the “free” massless Weyl fermions, with *λ* = ±1 the band index and *ξ* = ±1 their chirality. In addition, the retarded self-energy has the form8
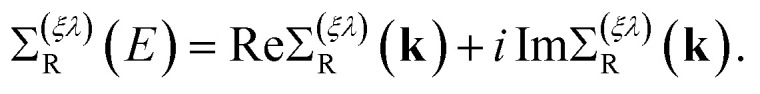


As usual, the real part of the self-energy renormalizes the single-particle energy spectrum, while the imaginary part represents the *scattering relaxation time τ*^(*ξλ*)^(*k*) through the relation9
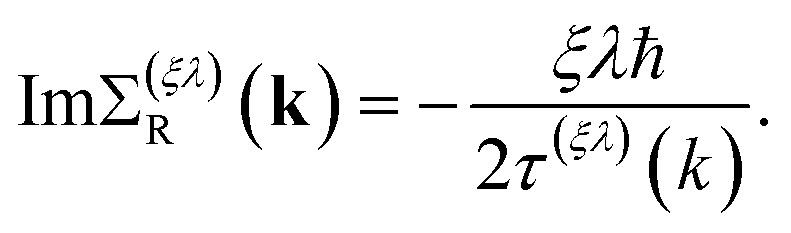


The advanced self-energy is given by the complex conjugate of the retarded self-energy, *i.e.*, *Σ*^(*ξλ*)^_A_(*E*) = [*Σ*^(*ξλ*)^_R_(*E*)]*. Similarly, the advanced Green's function is given by 
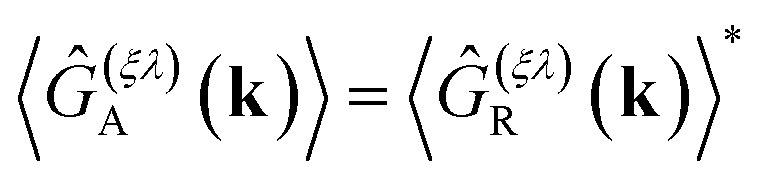
.

As discussed in standard ref. 40 and 41, for small concentrations *n*_d_/(π*k*_F_^2^) < 1, the total Green's function in eqn (7) can be accurately calculated by adding the sequence of diagrams for the retarded self-energy as presented in ref. 37, an approach known as the non-crossing approximation (NCA). This series of diagrams corresponds to the configurational average of the *T*-matrix over the random distribution of dislocations after eqn (6)10
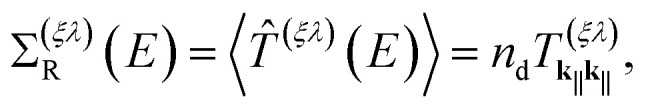
where the elements of the *T*-matrix are given by 
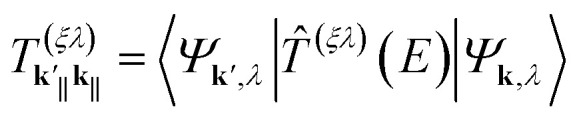
 and **k** = (**k**_‖_, *k*_*z*_), with **k**_‖_ = (*k*_*x*_, *k*_*y*_) the momentum on the plane perpendicular to the cylindrical dislocation axis. As we showed in ref. 37, the real part of the self-energy is expressed in terms of the phase shifts *δ*_*m*_(*k*) for each angular momentum component *m* ∈ *ℤ*11
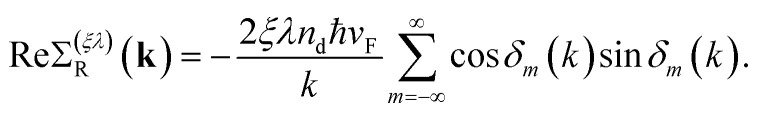


This infinite series over highly oscillatory terms converges to zero, and therefore no contribution arises from the real part of the self-energy. On the other hand, the imaginary part of the self-energy in eqn (9) gives the scattering relaxation time in terms of the phase shifts12
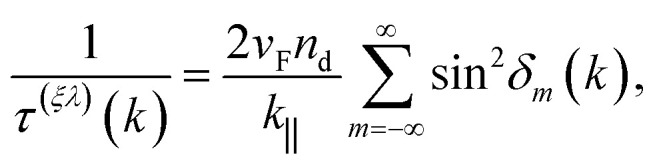


and we can see that it is a positive definite quantity, inversely proportional to the concentration of dislocations *τ*^(*ξλ*)^ ∼ *n*_d_^−1^. The phase shifts *δ*_*m*_(*k*) for this system were calculated in ref. 35 and their analytical expression is given in eqn (1) of the ESI.† Also, the explicit expression for the *T*-matrix elements in terms of these phase shifts is given in eqn (2) of the ESI.†

## 3 Onsager coefficients in the linear response regime

In the present work, our purpose is to study the thermal and thermoelectric transport coefficients in these topological materials in the presence of a finite concentration of dislocations *n*_d_ as the single scattering mechanism. For this purpose, in what follows we shall apply the basic principles of non-equilibrium thermodynamics. Associated with the particle current **ĵ**, defined by the operator13
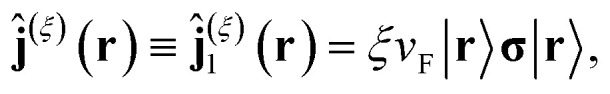


and the heat current operator **j**^(*ξ*)^_*Q*_14
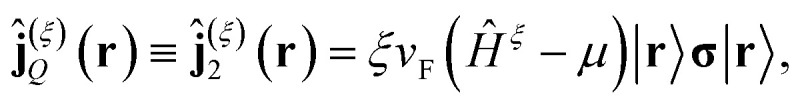


the macroscopic currents are given by the corresponding ensemble averages15
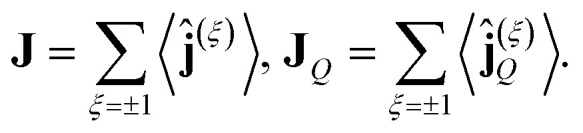


The entropy production rate is expressed in terms of the macroscopic currents and gradients as follows^41,42^16
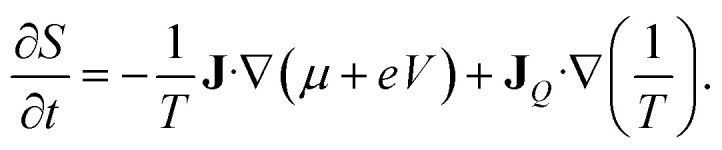


Let us introduce the Onsager coefficients by means of the tensor notation17a
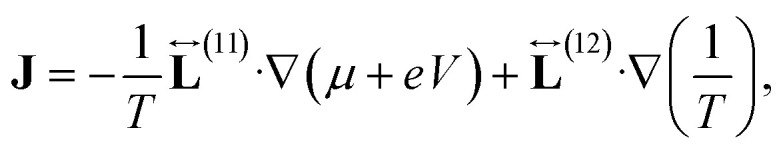
17b
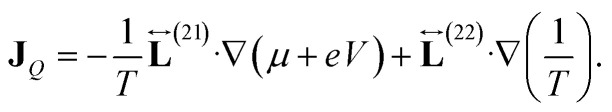


The transport coefficients can also be expressed in terms of these tensors, by applying the corresponding definition as follows: first, let us assume that ∇*T* = 0 and ∇*μ* = 0, such that the electrical conductivity tensor is then given by18
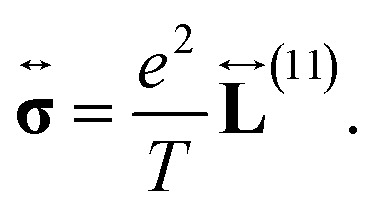


On the other hand, we remark that the thermal conductivity, by definition, is measured under conditions such that no electric current flows through the material **J** = 0. Then, combining eqn (17a) and (17b) we conclude that the thermal conductivity tensor is given by the expression19
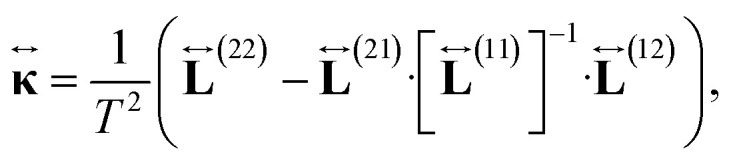


while the Seebeck coefficient (thermopower) is given by20
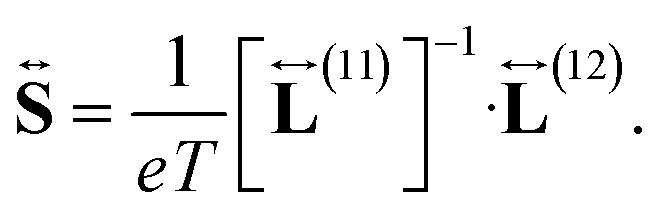


The Onsager coefficients can be expressed in terms of the Kubo formulae in the linear response regime. From the entropy production rate in eqn (16), we have21
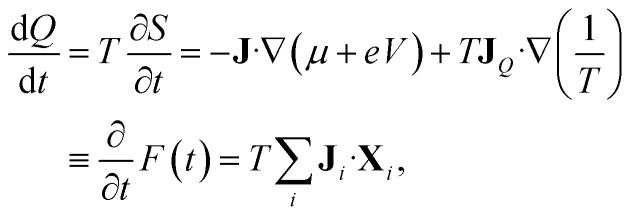
where **J**_1_ = **J**, **J**_2_ = **J**_*Q*_, and22
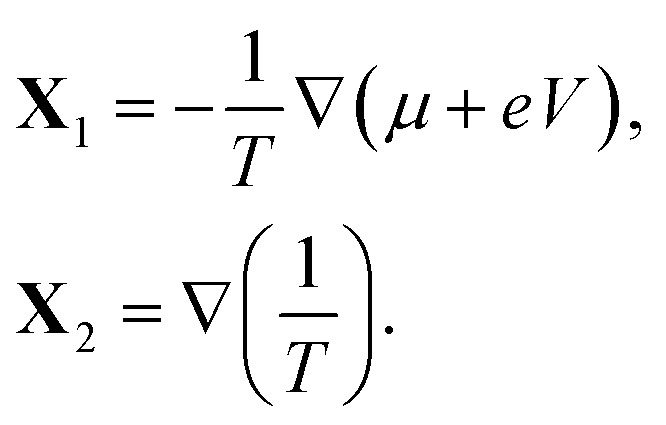


In eqn (21), *F*(*t*) is usually termed the “dissipation function”.^41^

We shall apply the Luttinger formalism^43^ for the evaluation of the Onsager coefficients. For this purpose, we begin by expressing the Kubo formulae for the different currents (*i* = 1, 2) in the form23
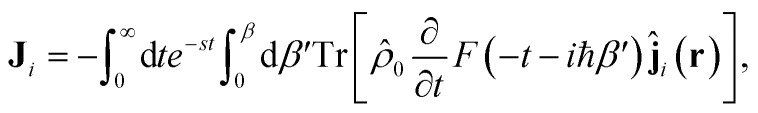
where *s* is a positive quantity that guarantees the adiabatic switching-on of the perturbation that drives the system out of equilibrium, and the limit *s* → 0^+^ is taken at the end of the calculation. In eqn (23), we also defined the equilibrium density operator24
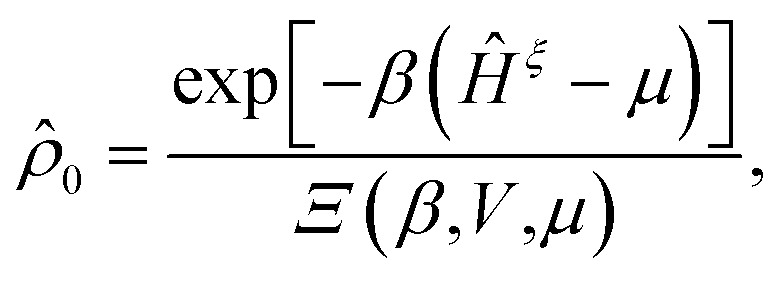
where *Ξ*(*β*,*V*,*μ*) = Tr exp[−*β*(*Ĥ*^*ξ*^ − *μ*)] is the grand-canonical partition function.

When inserting eqn (21) into eqn (23), we obtain25
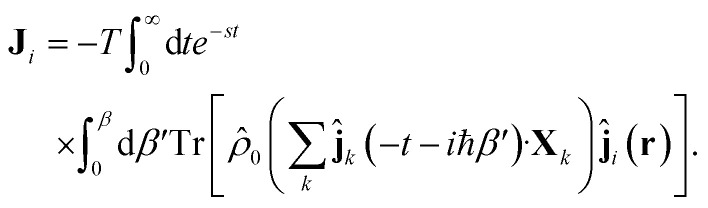


Then, we conclude26
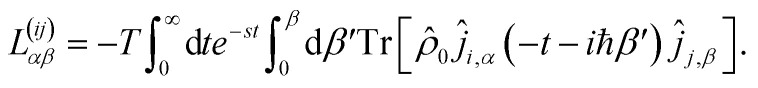


In these expressions, the electric eqn (13) and heat current eqn (14) operators, respectively, can be combined into a single definition (for *i* = 1, 2)27
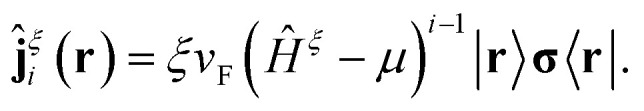


Finally, as we show in Section 3 of the ESI,† we obtain the corresponding Onsager coefficients by considering the spatial average of the corresponding tensors. This is equivalent, in Fourier space, to take the limit of the momentum **q** → 0 in each of these coefficients28
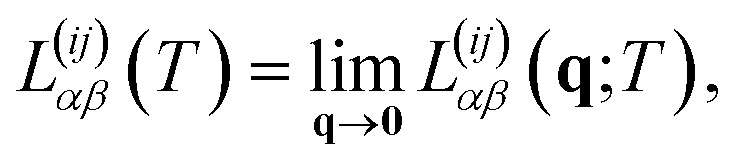
where 
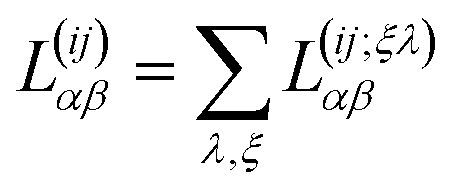
 involves the linear superposition of band and chiral components.

In particular, in the limit of low concentrations *n*_d_/π*k*_F_^2^ < 1, the Onsager coefficients (for *i*, *j* = 1, 2) are given by29
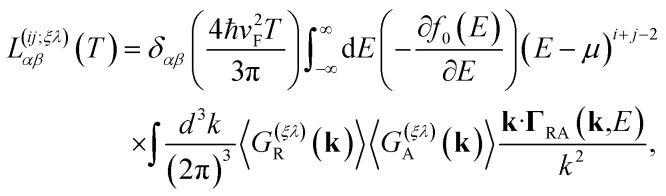
where we have taken into account the vertex corrections **Γ**_RA_(**k**, *E*), as described in Section 2 of the ESI.†

At low temperatures, a closed analytical solution is possible since the derivative of the Fermi distribution takes a compact support at the Fermi energy. Therefore, we can evaluate the vertex function at the Fermi momentum *k*^*ξ*^_F_, to obtain for the bulk Onsager coefficients the simplified expressions (for *i*, *j* = 1, 2)30
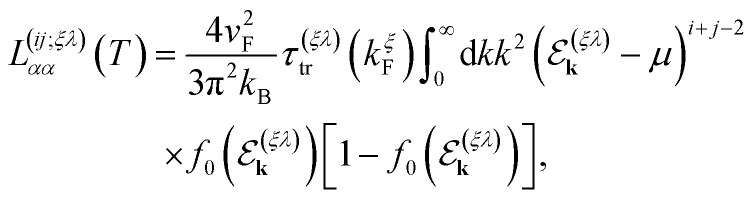
where the total *transport relaxation time*, *τ*^(*ξλ*)^_tr_(*k*^*ξ*^_F_), is given by31
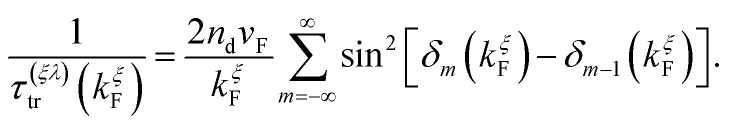


We remark that, along with the scattering relaxation time derived directly from the self-energy in eqn (12), the transport relaxation time in eqn (31) is inversely proportional to the concentration of dislocations *τ*^(*ξλ*)^_tr_ ∼ *n*_d_^−1^. The details of its derivation, as well as the computation of the integrals in eqn (30), are described in detail in the ESI.† In terms of these integrals, we finally obtain closed analytical formulae for the Onsager coefficients:32
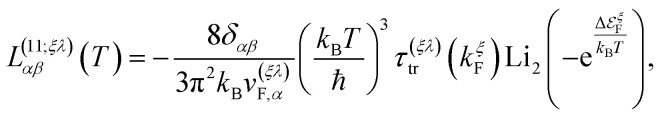
33
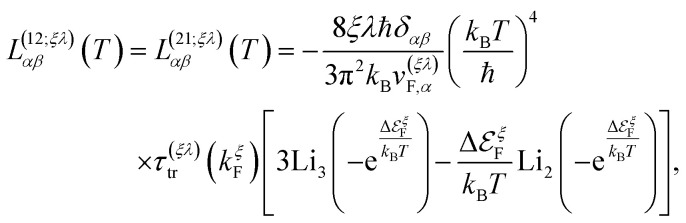
and34
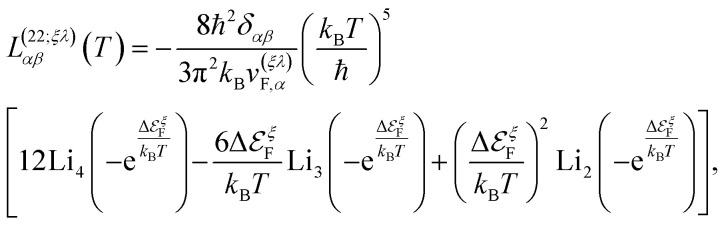
where in all those expressions, Li_*s*_(*z*) stands for the polylogarithm function of order *s*. The electrical conductivity is obtained from eqn (18) and (32)35
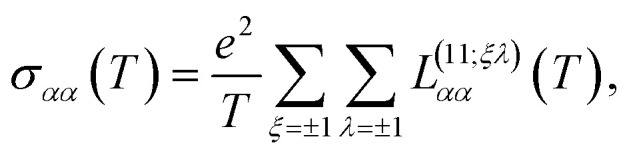


and then, we obtain36
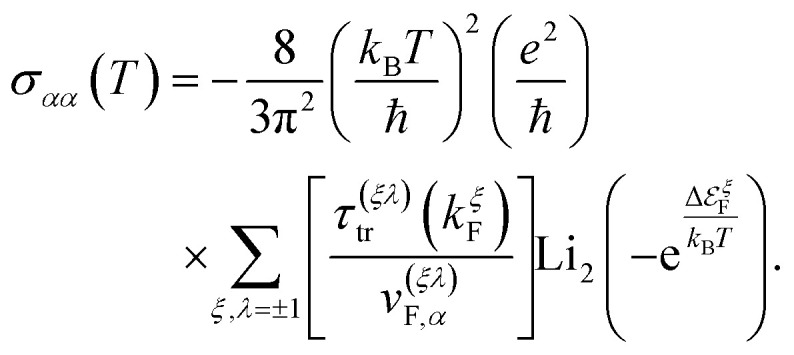


Similarly, the electronic thermal conductivity is obtained from eqn (19) and (32)–(34)37
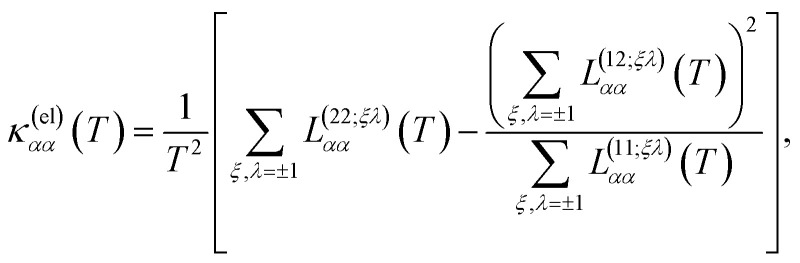


and the Seebeck coefficient is obtained from eqn (20), (32) and (33)38
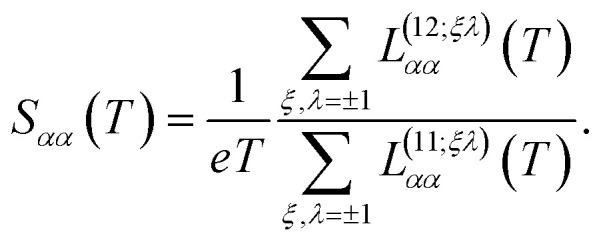


## 4 Results

In this section, we shall evaluate our analytical expressions to estimate the transport coefficients of several WSMs in the family of transition metal monopnictides. For this purpose, we shall consider the microscopic/atomistic parameters obtained from first-principles calculations, as reported in ref. 39 and 17. We shall also take into account the anisotropies reported by ref. 17 in the Fermi velocities and density of charge carriers at different Weyl nodes (*ξ* = ±) and bands (*λ* = ±), respectively. These two references computed values for the Fermi energy with respect the position of each of the Weyl nodes as presented in Table 2.

**Table 2:** Values of 
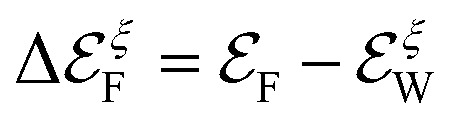
 from *ab initio* computations reported in the literature. Here, 
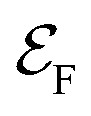
 is the Fermi level and 
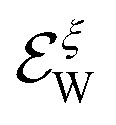
 is the energy at the Weyl node *ξ*. We use the average of the two values

Material	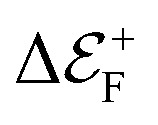 (eV)^17^	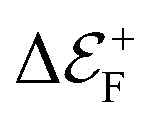 (eV)^39^	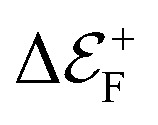 (eV) average	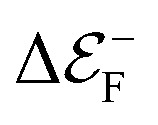 (eV)^17^	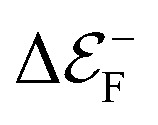 (eV)^39^	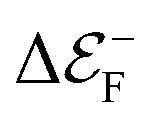 (eV) average
TaAs	0.026	0.0221	0.02405	0.013	0.0089	0.01095
TaP	0.055	0.0531	0.05405	−0.021	−0.0196	−0.0203
NbAs	0.033	0.0322	0.0326	−0.004	−0.0042	−0.0041
NbP	0.056	0.0534	0.0547	−0.026	−0.0259	−0.02595

We shall assume that the *z*-direction is aligned with the crystallographic direction of the defect axes, while the temperature and/or voltage gradients are imposed parallel to the *xy*-plane. Therefore, we shall employ the *x*- and *y*-components of the Fermi velocities *v*^(*ξλ*)^_F,*α*_ averaged from the reported values given in ref. 17 and 39 (see Table 3), for the conduction (*λ* = +1) and valence (*λ* = −1) bands, as well as for each of the chiral Weyl nodes (*ξ* = ±1), respectively. From the energies presented in Table 2 and the Fermi velocities given in Table 3, we can compute the Fermi momenta at each node using the formula given in ref. 17.39
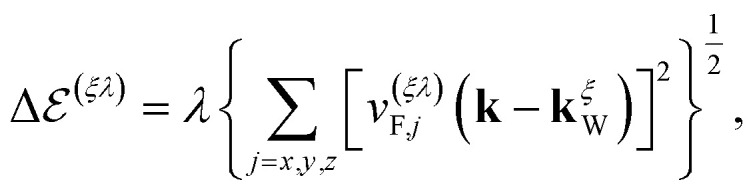
where **k**^*ξ*^_W_ is the wave-vector location of each Weyl node in momentum space. The computed values of the Fermi momenta are shown in Table 4.

**Table 3:** Values of the Fermi velocity *v*^(*ξλ*)^_F,*α*_ in the units of 10^5^ m s^−1^. In the valence band (*λ* = −1) they correspond to hole velocities. We use the average of the reported values in ref. 17 and 39. Tables with the reported values are given in Section 4 of the ESI

Material	*v* ^(++)^_F,*x*_	*v* ^(+−)^_F,*x*_	*v* ^(−+)^_F,*x*_	*v* ^(−−)^_F,*x*_	*v* ^(++)^_F,*y*_	*v* ^(+−)^_F,*y*_	*v* ^(−+)^_F,*y*_	*v* ^(−−)^_F,*y*_	*v* ^(++)^_F,*z*_	*v* ^(+−)^_F,*z*_	*v* ^(−+)^_F,*z*_	*v* ^(−−)^_F,*z*_
TaAs	2.85	−5.25	2.5	−4.3	2.2	−2.3	3.5	−1.75	0.2	−0.2	4.35	−1.6
TaP	3.4	−5.55	2.15	−4.0	2.55	−2.55	3.05	−2.05	0.2	−0.2	4.3	−1.45
NbAs	2.75	−4.8	2.45	−3.25	1.65	−1.7	2.3	−1.25	0.1	−0.1	3.65	−1.15
NbP	3.35	−5.4	1.9	−2.8	2.2	−2.3	2.05	−1.65	0.0(3)	−0.0(3)	4.0	−1.2

**Table 4:** Values of *k*^*ξ*^_F_ computed from the average 
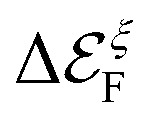
 given in Table 2 and the Fermi velocities given in Table 3 using eqn (39)

Material	*k* ^+^_F_ (nm^−1^)	*k* ^−^_F_ (nm^−1^)
TaAs	0.1013	0.0272
TaP	0.1796	0.0653
NbAs	0.1544	0.0170
NbP	0.2073	0.1138

In order to estimate the geometric and structural parameters involved in the model, we follow the analysis presented in our previous work.^35^ Therefore, we assume that the cylindrical regions representing the dislocations have a radius *a* = 15 nm. From the proportionality relation between the torsional angle *θ* (in degrees) and the pseudo-magnetic field representing strain |**B**^*ξ*^|*a*^2^ = 1.36*θφ̃*_0_,^35^ the modified flux quantum associated with the dislocations in these materials is approximately 
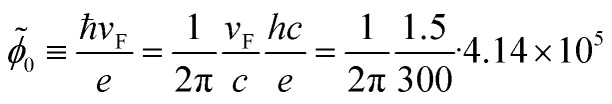
 T Å^2^ ≈ 330 T Å^2^. Moreover, for definiteness, in this work we have chosen a torsion angle *θ* = 15*°*. Finally, for the parameter *α* = *V*_0_/h*v*_F_ that captures the effect of the delta barrier representing the lattice mismatch at the edge of the cylindrical dislocation, we follow our previous estimations based on Frank's law^35^ by setting *α* = 3π/4.

As clearly seen in eqn (36)–(38), our analytical expressions for the electronic transport coefficients depend on the total transport relaxation time due to the scattering with the dislocations at the Fermi energy. In Table 5, we present calculated values of such relaxation times, for different transition metal monopnictides, assuming the structural parameters in our model. For an estimation of the concentration of defects *n*_d_ in real crystal systems, ref. 38 reports a native concentration of dislocations in the range *n*_d_ ∼ 10^5^ to 10^7^ cm^−2^ for the materials TiO_2_ and SrTiO_3_. These concentrations can be enhanced using different treatments up to 10^13^ cm^−2^, close to the rendering amorphous limit. Also, as is pointed out in ref. 44, the maximal practical density of screw dislocations detected in materials using electronic microscopy is in the range 10^11^ to 10^12^ cm^−2^. Assuming then that a realistic concentration of dislocations would be in the range *n*_d_ ∼ 10^9^ to 10^11^ cm^−2^, an important aspect to check is if the ratio *n*_d_/(π*k*_F_^2^) < 1 for the materials involved, in order for our approximations to be valid. From the values for the Fermi momenta reported in Table 4, we see that the four materials satisfy *k*^±^_F_ > 0.01 nm^−1^. Therefore, for the aforementioned range of concentrations, the ratio *n*_d_/(π*k*_F_^2^) ∼ 10^−3^ to 10^−1^, and hence our approximations are well justified for all four materials analyzed in this study.

**Table 5:** Transport relaxation time (along the *x*-direction) for each node *ξ* = ±1 and material. The result was computed from eqn (31) by assuming a concentration of dislocations *n*_d_ = 2 × 10^9^ cm^−2^

Material	*τ* ^+^_tr_ (10^−12^ s)	*τ* ^−^_tr_ (10^−12^ s)
TaAs	4.24	2.71
TaP	3.79	3.00
NbAs	4.64	2.16
NbP	3.90	5.55

**Fig. 2:**
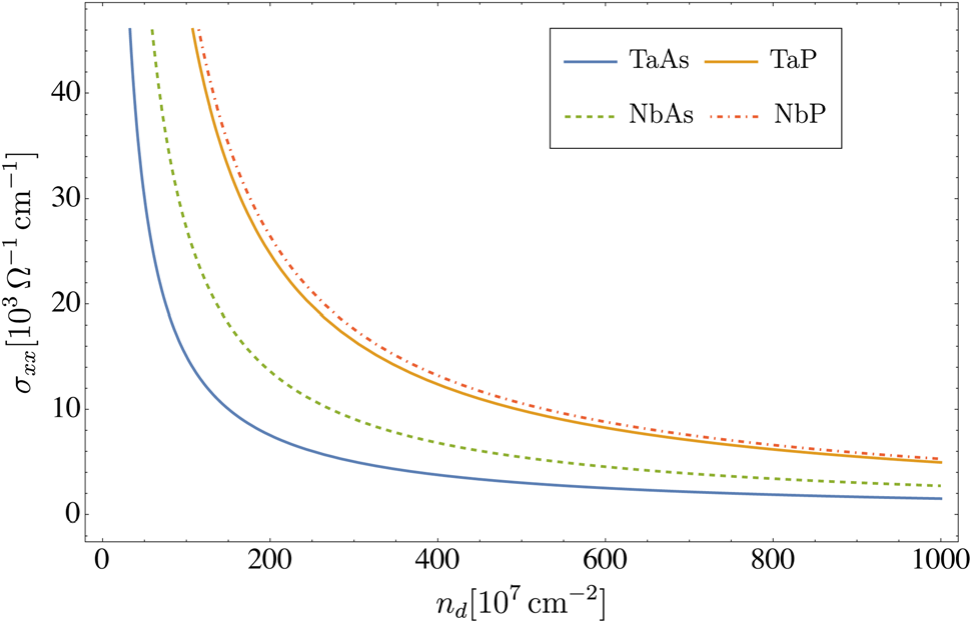
Electrical (DC) conductivity *σ*_*xx*_*versus* the concentration of dislocations computed from eqn (37) at *T* = 5 K, for the transition metal monopnictides TaAs, TaP, NbAs and NbP.

**Fig. 3:**
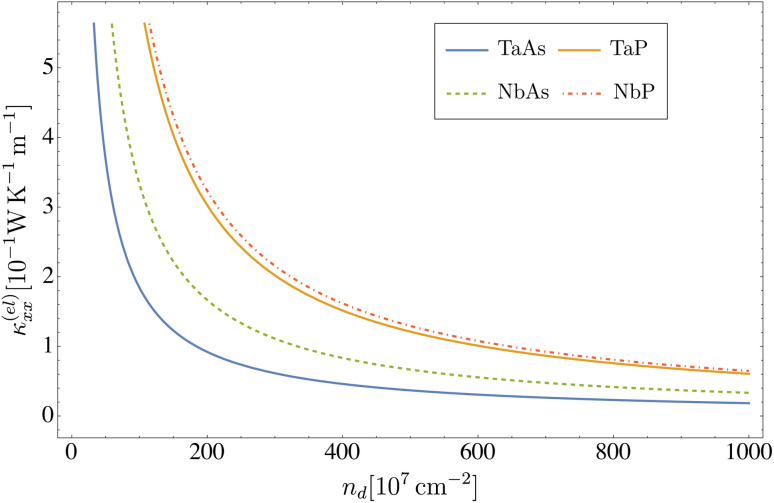
Electronic thermal conductivity *κ*^(el)^_*xx*_*versus* the concentration of dislocations computed from eqn (37) at *T* = 5 K, for the transition metal monopnictides TaAs, TaP, NbAs and NbP.

The DC conductivity and electronic thermal conductivity, as a function of the concentration of dislocations, are displayed in Fig. 2 and 3, respectively, where a temperature *T* = 5 K was assumed. We see that both transport coefficients exhibit an inverse proportionality, *i.e. σ*_*xx*_ ∼ *n*_d_^−1^ and *κ*_*xx*_ ∼ *n*_d_^−1^, since the transport relaxation time defined in eqn (31) is itself inversely proportional to the concentration of defects *τ*^(*ξλ*)^_tr_ ∼ *n*_d_^−1^. In particular, as seen in Table 5, for a concentration of dislocations *n*_d_ = 2 × 10^9^ cm^−2^ the relaxation times in all four materials are on the order of *τ*_tr_ ∼ 10^−12^ s.

**Fig. 4:**
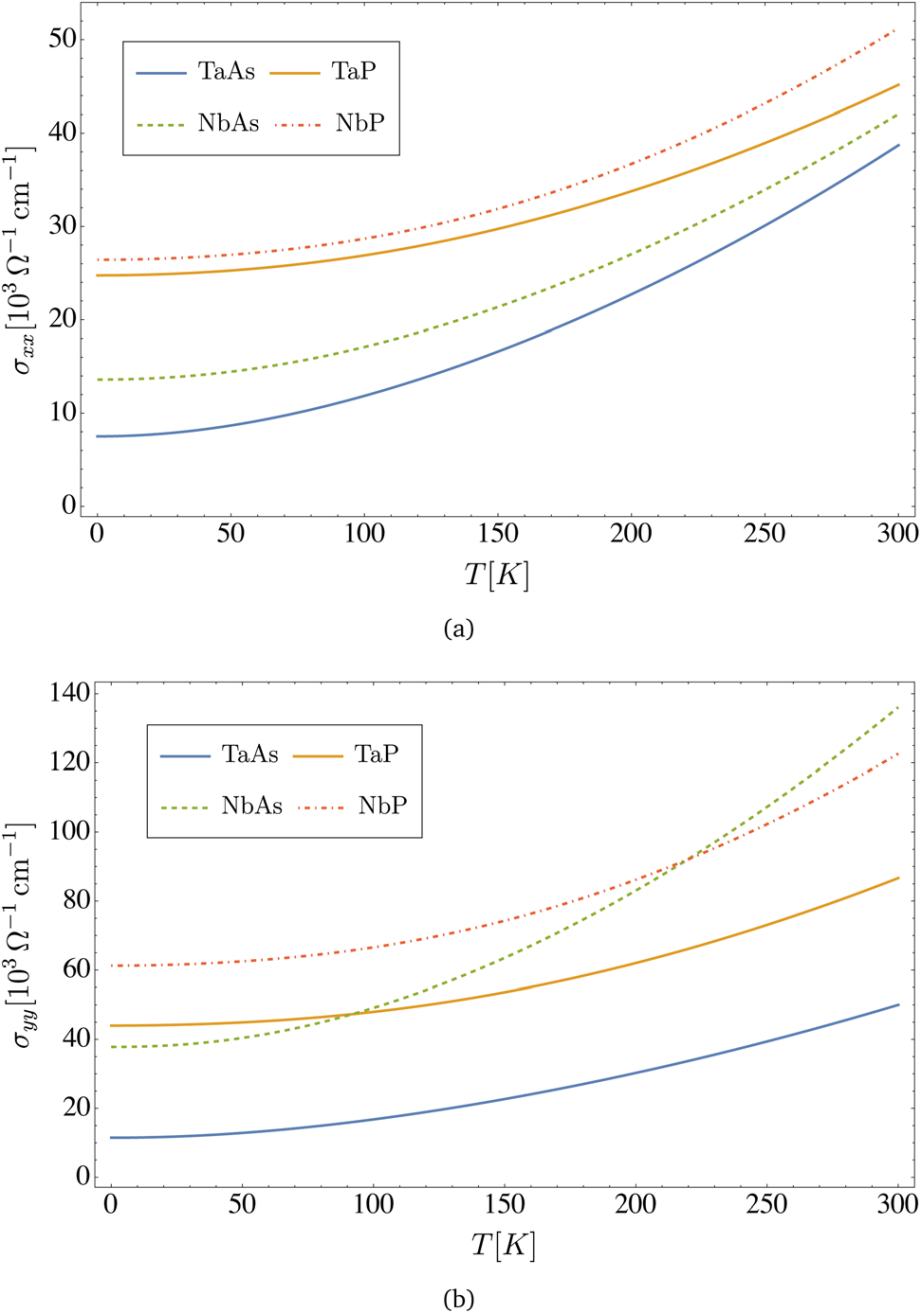
The figure shows the DC electrical conductivity for the transition metal monopnictides TaAs, TaP, NbAs and NbP: (a) displays *σ*_*xx*_ and (b) displays *σ*_*yy*_. Here we assume a concentration of dislocations of *n*_d_ = 2 × 10^9^ cm^−2^.

**Fig. 5:**
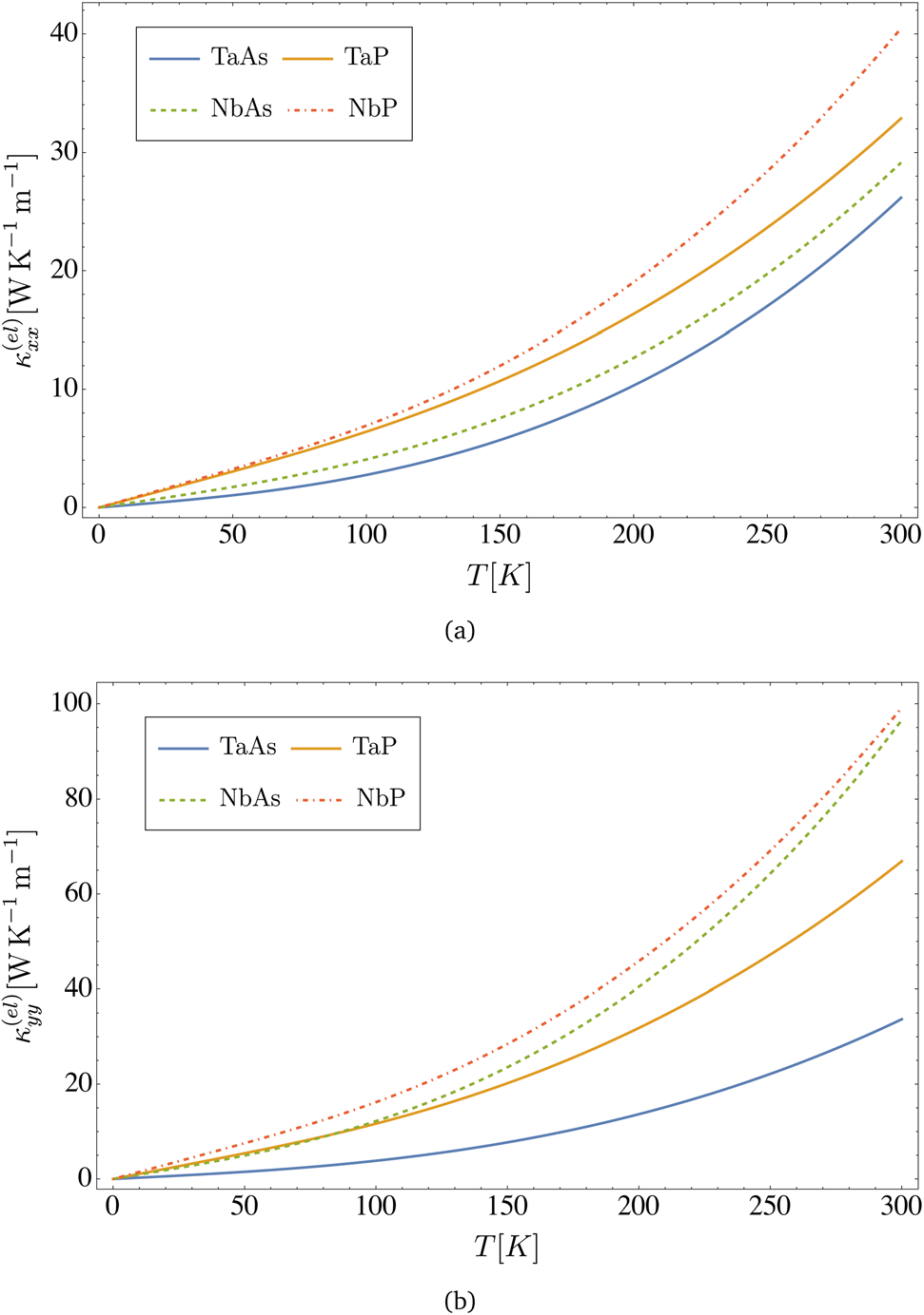
Electronic thermal conductivity *versus* temperature computed from eqn (37) for the transition metal monopnictides TaAs, TaP, NbAs and NbP: (a) shows *κ*^(el)^_*xx*_ and (b) shows *κ*^(el)^_*yy*_. Here we assume a concentration of dislocations of *n*_d_ = 2 × 10^9^ cm^−2^.

As displayed in Fig. 4, the electrical conductivity of all materials depends on temperature, and assuming a concentration of defects of *n*_d_ = 2 × 10^9^ cm^−2^, it is in the range of *σ*_*xx*_ ∼ 10^4^ to 10^5^ Ω^−1^ cm^−1^, with NbP being the better conductor. A similar hierarchy among the four materials is observed in Fig. 5 for the electronic thermal conductivity, which displays a nearly linear dependence up to room temperature. Results computed from eqn (36) for the DC conductivity along *x*- and *y*-directions at zero and room temperatures are presented in Table 6. We observe anisotropy between the *x*- and *y*-directions, due to the anisotropy in the components of the Fermi velocity, as can be appreciated in the values displayed in Table 3.

**Table 6:** Values of the *σ*_*xx*_ and *σ*_*yy*_ DC conductivities (in the units of 10^3^ Ω^−1^ cm^−1^) at 0 K and 300 K for each material. The result was computed from eqn (36) by assuming a concentration of dislocations *n*_d_ = 2 × 10^9^ cm^−2^

Material	*σ*_*xx*_ (0 K)	*σ*_*yy*_ (0 K)	*σ*_*xx*_ (300 K)	*σ*_*yy*_ (300 K)
TaAs	7.52	11.46	38.71	50.00
TaP	24.75	43.99	45.16	86.66
NbAs	13.61	37.80	42.05	136.08
NbP	26.42	61.26	51.24	122.62

As seen in Fig. 5, the room temperature electronic thermal conductivity in all four compounds, assuming the same concentration of defects *n*_d_ = 2 × 10^9^ cm^−2^, is on the order *κ*^(el)^_*xx*_ ∼ 10 to 10^2^ W K^−1^ m^−1^. The reason why NbP exhibits higher values of electrical and thermal conductivity as compared to the other materials in the monopnictide family is because its *k*_F_ is almost an order of magnitude larger, as shown in Table 4. The effect becomes dominant due to the presence of *k*_F_ in the exponential of the argument of the polylogarithmic functions in the analytical expressions for the electric and thermal conductivities. In physical terms, since such an exponential factor arises from the Fermi–Dirac distribution at finite temperature, it implies a higher population of chiral Weyl fermions available for transport as compared with the other materials in the same transition monopnictide family. Results calculated using eqn (37) for the electronic contribution to the thermal conductivity along *x*- and *y*-directions at room temperatures are presented in Table 7. Again, we observe anisotropy between the *x*- and *y*-directions, as a consequence of the anisotropy in the Fermi velocity components in these materials.

**Table 7:** Values of the *κ*^(el)^_*xx*_ and *κ*^(el)^_*yy*_ electronic thermal conductivities (in the units of W K^−1^ m^−1^) at 300 K for each material. The result was computed from eqn (37) by assuming a concentration of dislocations *n*_d_ = 2 × 10^9^ cm^−2^

Material	*κ* ^(el)^_*xx*_ (300 K)	*κ* ^(el)^_*yy*_ (300 K)
TaAs	26.17	33.68
TaP	32.88	66.90
NbAs	29.15	96.57
NbP	40.47	99.16

From the expressions for the electrical conductivity in eqn (36), the electronic thermal conductivity in eqn (37), and the Seebeck coefficient in eqn (38), we can compute the Lorenz number40
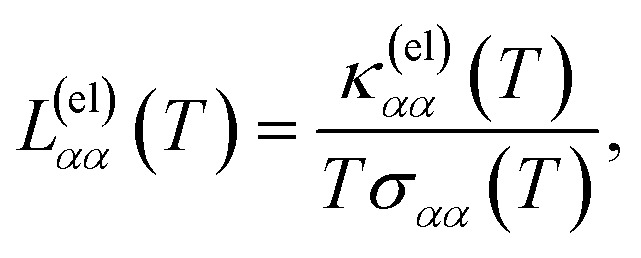


and the dimensionless figure of merit (based on the electronic thermal conductivity), an important indicator for thermoelectric applications41
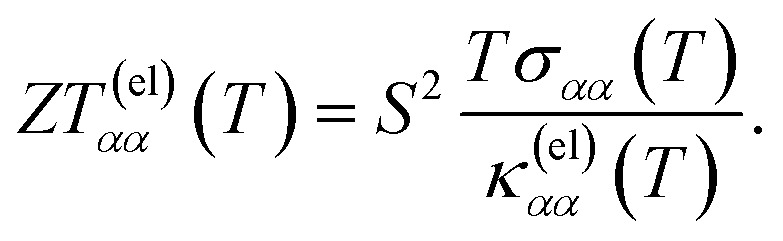


In Fig. 7, we represent the Lorenz number, calculated from eqn (40) for all four materials as a function of temperature. Remarkably, *L*(*T* → 0) → *L*_0_ = (π^2^/3)(*k*_B_/*e*)^2^, and hence the Wiedemann–Franz law is indeed satisfied in the limit of very low temperatures, a common feature for normal metallic systems, that is however also verified in these semimetal compounds.

**Fig. 6:**
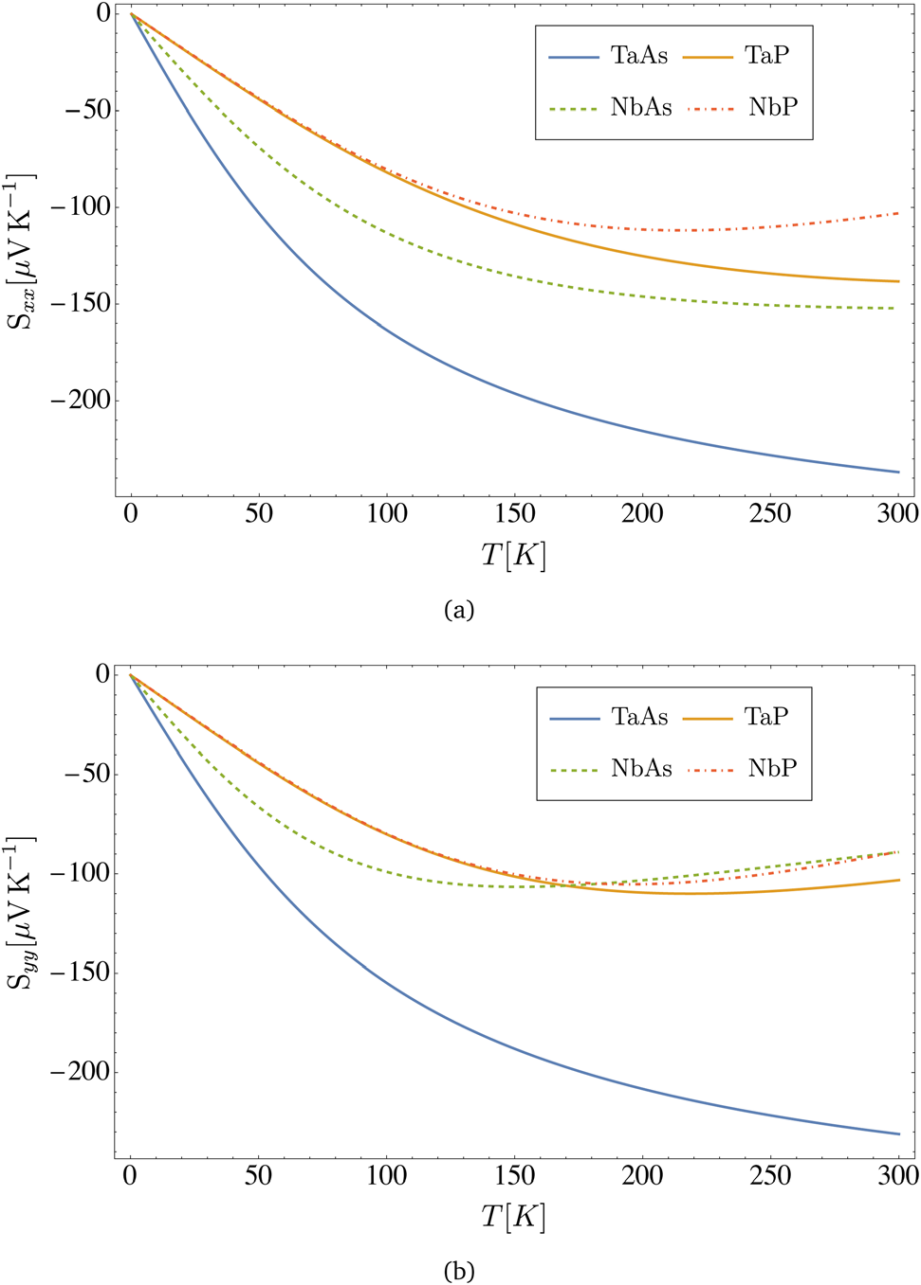
Seebeck coefficient *versus* temperature computed from eqn (38) for the transition metal monopnictides TaAs, TaP, NbAs and NbP: (a) shows *S*_*xx*_ and (b) shows *S*_*yy*_.

In Fig. 6, we present the Seebeck coefficient as a function of temperature, calculated from eqn (38) for the different materials. The negative sign of the Seebeck coefficient is consistent with the choice of a positive chemical potential, where the charge carriers are therefore electrons (instead of holes). For all materials, the Seebeck coefficient at room temperature is on the order of |*S*| ∼ 10^2^ μV K^−1^, and its absolute value grows at lower temperatures. This is consistent with different estimations in the literature for the family of transition metal monopnictides, reporting values in the range |*S*| ∼ 10^2^ to 10^3^ μV K^−1^ (ref. 23–25) at room temperature.

**Fig. 7:**
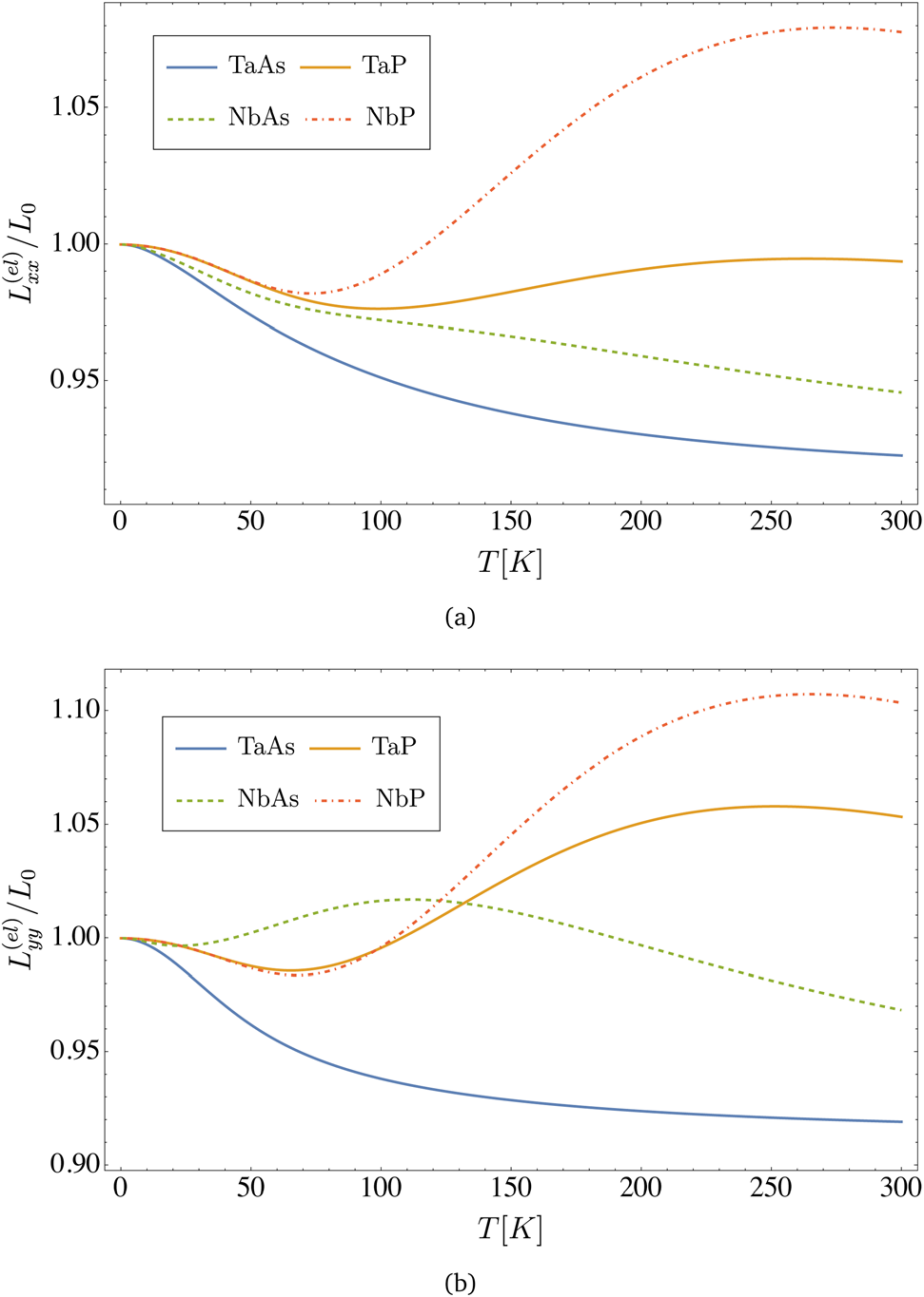
Electronic Lorenz number *versus* temperature computed from eqn (40) for the transition metal monopnictides TaAs, TaP, NbAs and NbP: (a) shows *L*^(el)^_*xx*_ and (b) shows *L*^(el)^_*yy*_. Notice that the value for the Wiedemann–Franz law is *L*_0_ = (π^2^/3)(*k*_B_/*e*)^2^ = 2.44 × 10^−8^ V^2^ K^−2^.

**Fig. 8:**
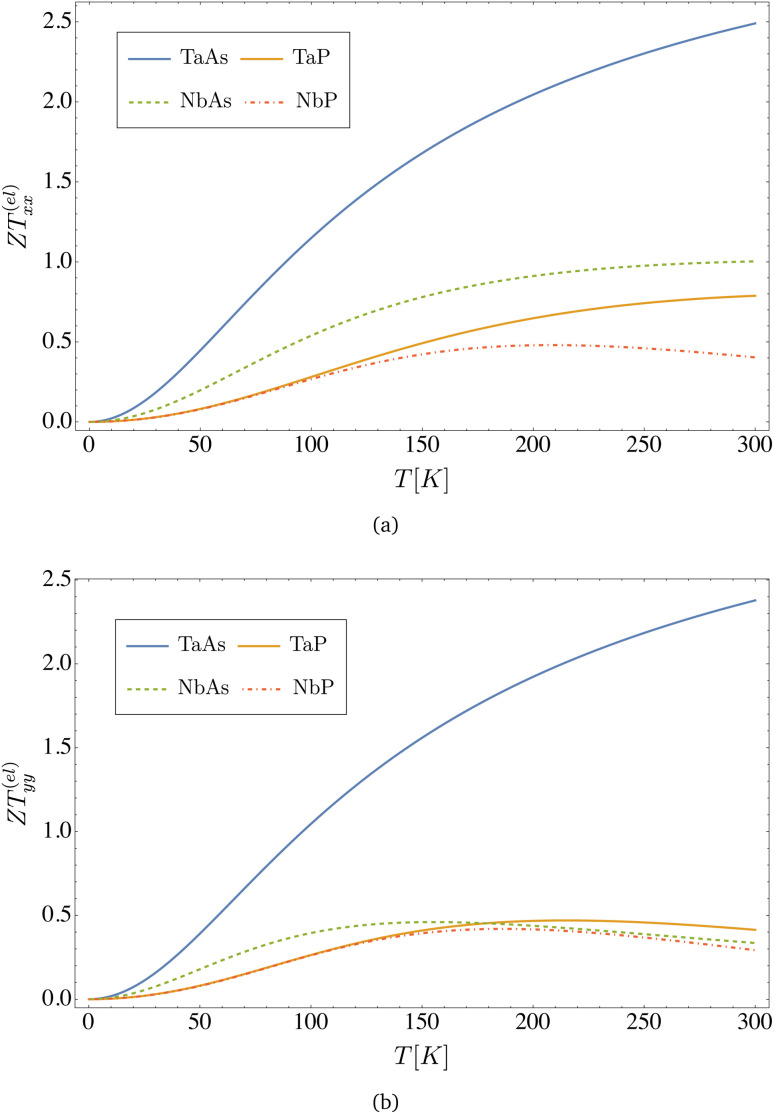
Electronic figure of merit *ZT*^(el)^ (dimensionless) *versus* temperature computed from eqn (41) for the transition metal monopnictides TaAs, TaP, NbAs and NbP: (a) shows *ZT*^(el)^_*xx*_ and (b) shows *ZT*^(el)^_*yy*_.

Finally, in Fig. 8 we present the figure of merit *ZT*^(el)^ calculated from eqn (41), for all different materials as a function of temperature. As both the DC conductivity and the electronic thermal conductivity are inversely proportional to the concentration of dislocations, *i.e. σ*_*xx*_ ∼ *n*_d_^−1^ and *κ*^(el)^_*xx*_ ∼ *n*_d_^−1^, this parameter cancels in their ratio in eqn (41), and hence *ZT*^(el)^ turns out to be independent of *n*_d_. However, a weak dependence on the presence of dislocations remains, since the scattering relaxation time is still a function of such defects through the scattering phase shifts *δ*_*m*_(*k*), as seen in eqn (31). Nevertheless, we could check that this effect also tends to cancel with the relaxation time upon taking the ratio leading to *ZT*^(el)^, and in practice this value becomes nearly independent on the presence of dislocations. Since according to Fig. 7 all four materials satisfy the Wiedemann–Franz law at very low temperatures *L*^(el)^(*T* → 0) → *L*_0_ = 2.44 × 10^−8^ V^2^ K^−2^, the low temperature limit of the figure of merit depends only on the Seebeck coefficient, *ZT*^(el)^(*T* → 0) ∼ *S*^2^(*T*)/*L*_0_, and hence it decreases to zero as *T* → 0, and increases with temperature as seen in Fig. 8 for all four materials. Remarkably, near room temperature, TaAs presents *ZT*^(el)^ > 2, which suggests that it could be an excellent candidate for thermoelectric applications. These findings are compatible with previous studies that proposed generic semi-metals for thermoelectric applications due to their relatively large Seebeck coefficients at room temperature |*S*| ∼ 10^2^ μV K^−1^,^26^ in agreement with the order of magnitude of our current estimations for the monopnictides, as well as with independent estimations for these compounds, reported in the literature^23–25^ to be in the range |*S*| ∼ 10^2^ to 10^3^ μV K^−1^ at room temperature.

## 5 Discussion

Our results for the electrical conductivity can be compared with independent estimations reported in the literature for the monopnictide family (TaAs, TaP, NbAs and NbP), where different scattering mechanisms where considered, particularly the electron–phonon interaction but not the dislocations studied in our work. The reported values, summarized in Table 1, show that the electrical conductivity is in the range *σ*_*xx*_ ∼ 10^4^ to 10^6^ Ω^−1^ cm^−1^, in agreement in the order of magnitude with our results in Fig. 4 for an estimated concentration of dislocations *n*_d_ = 2 × 10^9^ cm^−2^.

Our theoretical model is concerned with the role of scattering with the quenched distribution of dislocations, but it does not include other possible mechanisms, particularly the electron–phonon scattering. However, its contribution to the transport relaxation time may be significant as temperature increases enough to excite the relevant phonon modes. Moreover, the phonon spectrum itself can develop interesting topological features that may generate novel electron–phonon scattering mechanisms in WSMs, as discussed for instance in ref. 46. The latter is an entirely different mechanism, whose detailed analysis requires a separate model beyond the scope of the present work. The combination and competition between both scattering mechanisms can be estimated using Mathiessen's rule, such that the overall relaxation time including electron–phonon scattering would be^47^42
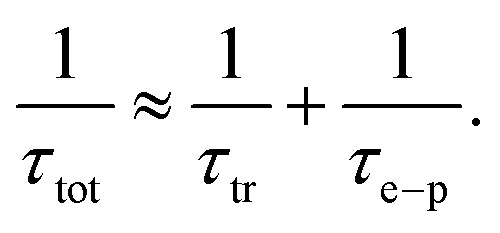


For instance, an estimation of the electron–phonon contribution is reported in ref. 18 and 25 as computed from first principles for TaAs and NbAs, and NbP respectively. The reported electron–phonon relaxation times at 300 K are on the order *τ*_e–p_ ∼ 10^−13^ seconds for all materials.^18,25^ In addition, ref. 23 reports an estimated value of the overall relaxation time (including electron–phonon, impurity and piezoelectric scattering) of *τ*_el_ = 3.01 × 10^−13^ at 300 K,^23^ which is still on the same order. In contrast, the calculated transport relaxation times for the scattering mechanism considered in this work, assuming a concentration of dislocations of *n*_d_ = 2 × 10^9^ cm^−2^ and displayed in Table 5, are larger, on the order of 10^−12^ seconds. However, as shown explicitly in Fig. 2 and 3 for the transport coefficients, the corresponding values for the relaxation time are inversely proportional to the concentration of dislocations, and hence their relative importance in comparison with other possible scattering mechanisms is strongly determined by this sample-dependent parameter. On the other hand, electron–phonon scattering is strongly dependent on temperature, and hence as an estimation we can interpolate it from its reported value *τ*_e–ph_(300) at 300 K, using the common Bloch-Gruneisen expression,^47^
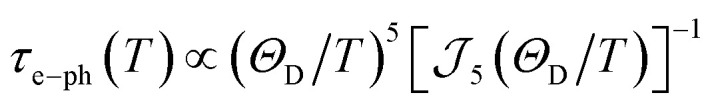
 such that43
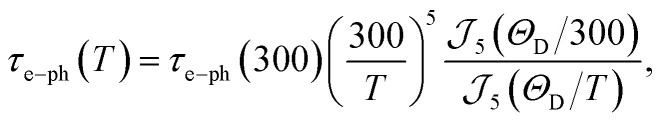


with *Θ*_D_ the Debye temperature and the function^47^44
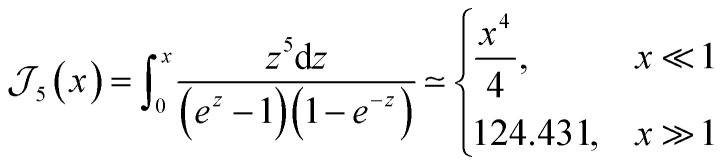


**Fig. 9:**
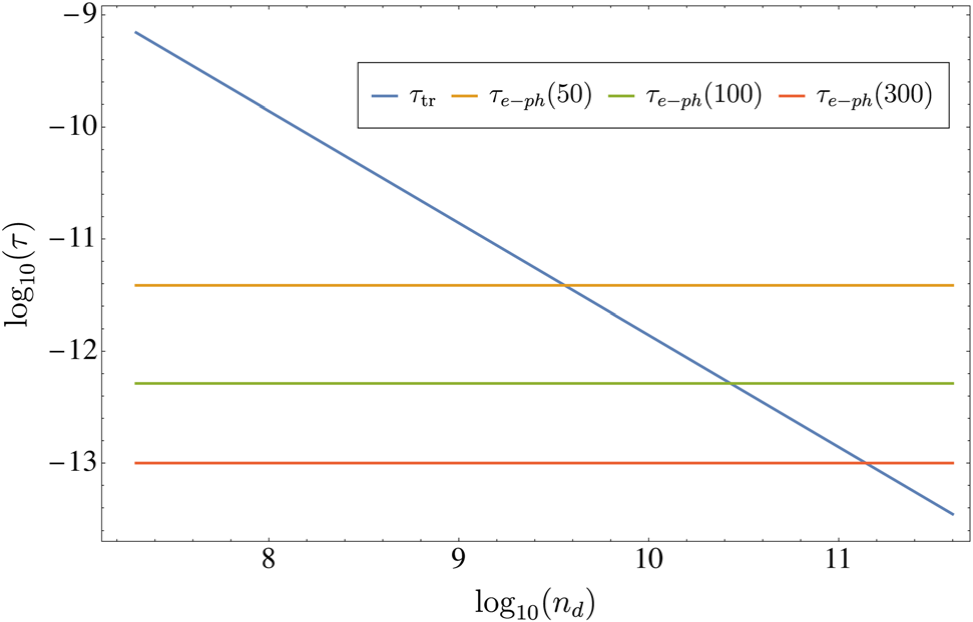
The figure shows the logarithm of the relaxation time *τ* (in seconds) *vs.* the logarithm of the concentration of dislocations *n*_d_ (in cm^−2^) for the material TaAs. Horizontal lines correspond to the values of *τ*_e–ph_(*T*) for three different temperatures. The value at *T* = 300 K is *τ*_e–ph_(300) ∼ 10^−13^ seconds.^18^ The values at *T* = 50 K and *T* = 100 K were computed using eqn (43), where *Θ*_D_ = 352 K is the experimental Debye temperature reported for TaAs.^45^

In Fig. 9, we represent the transport relaxation time *τ*_tr_ (solid blue line) for the scattering with dislocations studied in this work, as a function of their concentration *n*_d_, exhibiting the expected inverse proportionality. For the sake of comparison, we also present as solid horizontal lines the values for the electron–phonon scattering relaxation time at three different temperatures *τ*_e–p_(*T*), estimated from eqn (43). As can be seen in Fig. 9, at 300 K the scattering due to dislocations dominates over electron–phonon at concentrations *n*_d_ > 3 × 10^11^ cm^−2^, while the corresponding concentration threshold is given by *n*_d_ > 2 × 10^10^ cm^−2^ at *T* = 100 K, and *n*_d_ > 6 × 10^10^ cm^−2^ at *T* = 50 K, respectively.

Concerning the thermal conductivity, for the sake of comparison, ref. 23 reports a first principles calculation for the electronic contribution to the thermal conductivity of TaAs, including electron–phonon scattering but no dislocations as in this work. Their result at 300 K is *κ*^(el)^_*xx*_ = 56.87 W K^−1^ m^−1^, which is within the range of our calculated values as displayed in Fig. 5 and in Table 7, even when taking into account the anisotropy in the *x*- and *y*-directions already discussed.

Since the contribution from the overall relaxation time tends to cancel when taking the ratio of the transport coefficients in eqn (40), the Lorenz number obtained when other scattering mechanisms are present should still be close to our calculation. Indeed, ref. 23 also reports a value for the Lorenz number *L*^(el)^_*xx*_ = 2.27 × 10^−8^ V^2^ K^−2^, which is a small deviation from the Wiedemann–Franz law, in agreement with our results in Fig. 7.

Finally, in Fig. 8, we display the figure of merit *ZT*^(el)^ calculated from eqn (41), for all different materials as a function of temperature. At room temperature, NbAs and TaAs exhibit a comparatively large figure of merit, with *ZT*^(el)^_*xx*_ ∼ 2.5 for TaAs, suggesting that they could be excellent candidates for thermoelectric applications. We remark that, upon including the phonon contribution to the total thermal conductivity *κ*_Tot_ = *κ*^(el)^_*xx*_ + *κ*^(*l*)^_*xx*_, this value will decrease. Indeed, a crude estimation of this effect may be introduced using the formula45
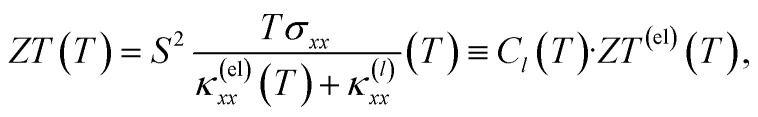
where we defined the correction factor due to the presence of the lattice conductivity by

46 *C*_*l*_(*T*) = (1 + *κ*^(*l*)^_*xx*_/*κ*^(el)^_*xx*_)^−1^.

However, as can be appreciated in Table 1, at 300 K the lattice thermal conductivities reported in the literature are in general smaller than their electronic counterparts, with the exception of TaP, and thus the correction factor is not far from unity for most cases. Considering our calculated values for the electronic thermal conductivity (assuming *n*_d_ = 2 × 10^9^ cm^−1^) at 300 K presented in Table 7, along with the values for the lattice thermal conductivity reported in the literature at the same temperature as displayed in Table 1, we have that for TaAs *C*_*l*_ = (1 + 36.06/26.17)^−1^ = 0.42, while for NbAs *C*_*l*_ = 0.96, indicating that even when including the phonon effects, the figure of merit for those two compounds *ZT* ∼ *C*_*l*_ × *ZT*^(el)^ ≥ 1 is still comparatively large, thus suggesting that they could be very attractive for thermoelectric applications. Moreover, as the presence of the torsional dislocations studied in this work will affect the mechanical properties of the lattice, thus enhancing phonon–phonon scattering, the lattice thermal conductivity will decrease in the presence of such defects as compared with the literature values quoted in Table 1. Therefore, our values for the figure of merit may actually be closer to reality than our corrected estimations here, but a detailed analysis of phonon effects is a matter for a separate study. As a final comment, we remark that no inter-valley scattering is involved in our analysis, for two main reasons. First, since the two valleys are well separated in momentum space, in order to couple them the potential scattering term must involve momentum exchange at least of this order of magnitude. On the other hand, in our formulation of the scattering problem across a single dislocation, the scattering term arises from the elastic gauge field connection at the same valley as the spinor state being scattered, and hence this constitutes a kinematic constraint preventing inter-valley scattering. Nonetheless, the possibility for inter-valley scattering cannot be ruled out completely, since other mechanisms besides the ones considered in this work may be in place.

## 6 Conclusions

Along this article, we have presented a theoretical analysis for thermoelectric transport coefficients in the family of transition metal monopnictides, when the sole scattering mechanism considered is the presence of a uniform, diluted concentration of torsional dislocation defects. Our approach is based on a combination of Green's functions with a statistical average over the random distribution of defects, leading to a Dyson equation with a self-energy in the non-crossing approximation, enhanced with vertex corrections. Moreover, from the analytical expressions for the retarded and advanced Green's functions, by means of general Onsager relations in non-equilibrium thermodynamics and the Luttinger formalism to implement the Kubo formulae, we obtained explicit analytical expressions for the electrical conductivity, thermal conductivity (electronic contribution) and Seebeck coefficient. Our analytical expressions are fairly general, and complemented with geometrical and microscopic parameters obtained from *ab initio* calculations, we could evaluate them to estimate the corresponding values of those transport coefficients for each material as a function of temperature and concentration of dislocations. This work provides a first step towards a theoretical analysis of transport in these systems, and hence we shall not delve into details concerning the experimental challenges posed, for instance, by the control of the sign of the charge carriers by a specific doping mechanism. Indeed, experimental and *ab initio* studies suggest that both electron and hole pockets will in general participate in transport.^48^ However, recent experimental studies^49^ on WSM films reveal that the presence of grain boundaries (that may play a similar role to our dislocation edges here) favours spontaneous predominance of holes (positive charge carriers) with a very high mobility, thus suggesting that doping may be achieved by defect engineering in these materials.

As presented in the Results and discussions sections, our analytical results predict values for the transport coefficients which are close to those reported in the literature, where different scattering mechanisms than this one are considered, particularly the electron–phonon interaction. For this particular case, we provided quantitative estimations of the range of temperatures and concentrations where each mechanism may become dominant. Finally, we remark that our results indicate that a high figure of merit is expected for at least two compounds in the family of transition metal monopnictides, *i.e.* TaAs and NbAs, even though our analytical expressions do not include the contribution from the lattice thermal conductivity. Nevertheless, based on reported values for this parameter in the literature, we estimate the order of the correction to our theoretical results to include the lattice effects, leading us to conclude that both TaAs and NbAs could be attractive candidates for thermoelectric applications. In this direction, we remark that the scattering mechanism analyzed in this work, *i.e.* the presence of torsional dislocation defects, introduces a moderate (as compared to electron–phonon) effect on electronic transport, whereas it may generate a strong phonon scattering mechanism decreasing the lattice thermal conductivity. Therefore, to engineer the concentration of such torsional dislocation defects in these materials, by decreasing the lattice thermal conductivity while nearly preserving the values of the electrical conductivity and Seebeck coefficient, may lead to even higher figures of merit than predicted here. However, an accurate assessment of the phonon transport mechanisms involved in this case goes beyond the scope of the present work and is a matter of outgoing research.

## Author contributions

Daniel A. Bonilla and Enrique Muñoz contributed equally to this paper.

## Conflicts of interest

There are no conflicts to declare.

## Supplementary Material

NA-006-D4NA00056K-s001
