# Supplementary material for: Thermoelectric transport in Weyl semimetals under a uniform concentration of torsional dislocations
Source: Nanoscale Adv. 2024 Apr 3;6(10):2701–12. doi: 10.1039/d4na00056k (PMC11093277; doi:10.1039/d4na00056k)
Supplement: NA-006-D4NA00056K-s001 [file NA-006-D4NA00056K-s001.pdf]

Electronic Supplementary Material (ESI) for Nanoscale Advances.
This journal is © The Royal Society of Chemistry 2024

Supplementary Material for "Thermoelectric transport in Weyl semimetals under a uniform concentration of torsional dislocations"

Daniel Bonilla [a] and Enrique Muñoz [a,b,*]

[a] *Facultad de Física, Pontificia Universidad Católica de Chile, Vicuña Mackenna 4860, Santiago, Chile.*

[b] *Center for Nanotechnology and Advanced Materials CIEN-UC, Avenida Vicuña Mackenna 4860, Santiago, Chile.*

[*] Correspondence: ejmunozt@uc.cl; Tel.: +56-2-2354-7625

1 Phase shifts

Here we reproduce the analytical expression for the phase shifts deduced in the supplemental material of Ref. [1]:

$$\tan\delta_m(k) = \frac{\lambda J_{m+1} - \varrho_n^{\xi} J_m \cdot z_a^{\frac{|m+1|-|m|}{2}} \frac{L_{n'_\rho}^{|m+1|}(z_a)}{L_{n_\rho}^{|m|}(z_a)} + \tan\alpha \left[J_m + \lambda\varrho_n^{\xi} J_{m+1} \cdot z_a^{\frac{|m+1|-|m|}{2}} \frac{L_{n'_\rho}^{|m+1|}(z_a)}{L_{n_\rho}^{|m|}(z_a)} \right]}{\lambda Y_{m+1} - \varrho_n^{\xi} Y_m \cdot z_a^{\frac{|m+1|-|m|}{2}} \frac{L_{n'_\rho}^{|m+1|}(z_a)}{L_{n_\rho}^{|m|}(z_a)} + \tan\alpha \left[Y_m + \lambda\varrho_n^{\xi} Y_{m+1} \cdot z_a^{\frac{|m+1|-|m|}{2}} \frac{L_{n'_\rho}^{|m+1|}(z_a)}{L_{n_\rho}^{|m|}(z_a)} \right]}. \tag{1}$$

Recall that δ_m is a function of k through the Bessel functions $Y_m \equiv Y_m(ka)$, $J_m \equiv J_m(ka)$, and $z_a = |B_\xi| a^2/2\tilde{\phi}_0$ (a is the radius of the cylindrical defect). The analytical expression for the T-matrix elements in terms of the phase shifts $\delta_m(k)$ for each angular momentum channel $m \in \mathbb{Z}$ are [2]

$$T^{(\xi\lambda)}_{\mathbf{k'}_\parallel \mathbf{k}_\parallel} = -\frac{2\xi\lambda\hbar v_F}{k} \sum_{m=-\infty}^{\infty} e^{i\delta_m(k)} \sin\delta_m(k) e^{im\phi}, \tag{2}$$

where ϕ is the scattering angle between $\mathbf{k}_\parallel$ and $\mathbf{k'}_\parallel$. Recall that $\mathbf{k}_\parallel = (k_x, k_y)$ are momentum vectors on the plane perpendicular to the dislocations' axis.

2 Vertex corrections

The self-energy contribution modifies the definition of the retarded and advanced Green's functions as depicted by the double lines in Fig. 1(b). However, there are also scattering processes involving links between the two internal Green function lines, as depicted in Fig. 1(a). When considering such diagrams with cross-links, as in Fig. 1(a), we must include the vertex correction as depicted in Fig. 1(b), where the vertex function

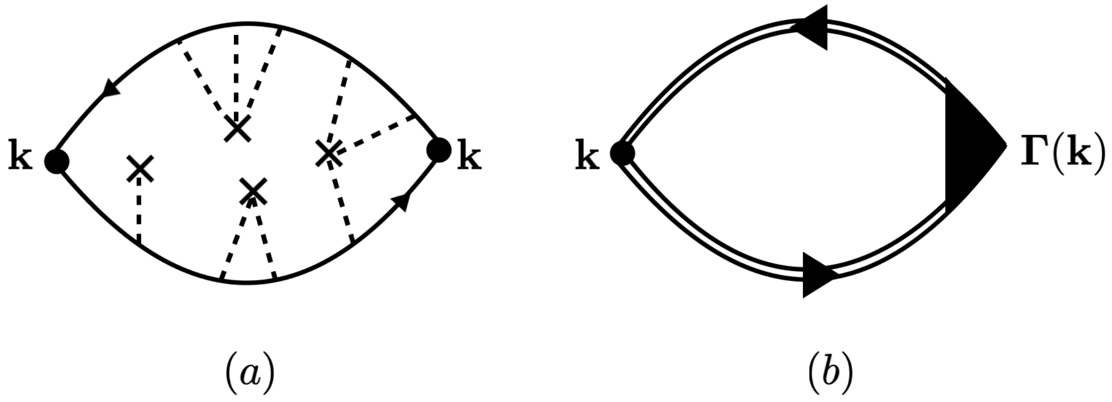

Fig. 1: (a) A typical diagram contributing to the Onsager coefficients in Eq (**??**), involving the configurational average of the two internal GF with cross-links between them. The upper line corresponds to the retarded GF and the lower to the advanced GF. (b) Diagrammatic representation of the two complete averaged GF (double lines) corresponding to the sum of all diagrams of the kind in (a) with the vertex correction $\boldsymbol{\Gamma}(\mathbf{k})$.

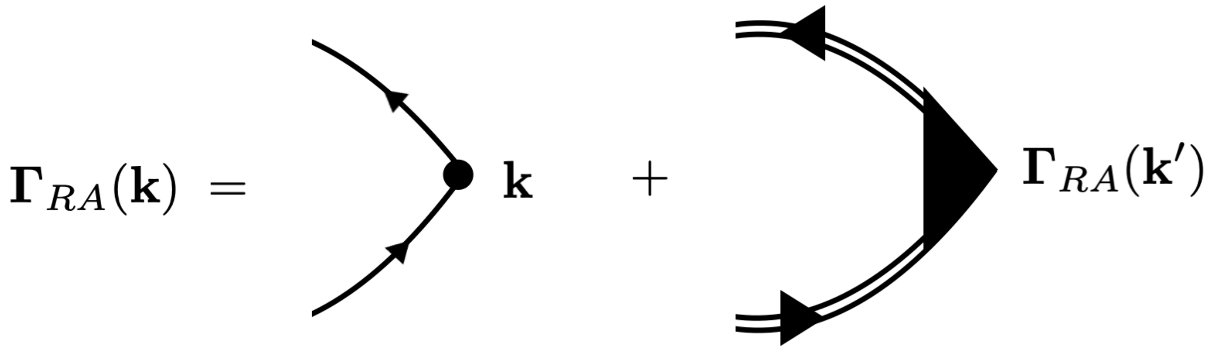

Fig. 2: The Bethe-Salpeter integral equation for the vertex function $\boldsymbol{\Gamma}_{RA}(\mathbf{k})$.

$\boldsymbol{\Gamma}_{RA}(\mathbf{k}, E)$ is the solution to the *Bethe-Salpeter equation* as depicted in Fig. 2. Then, we have

$$\boldsymbol{\Gamma}_{RA}(\mathbf{k}, E) = \mathbf{k} + n_d \int \frac{d^2 k'_\parallel}{(2\pi)^2} \left\langle G_R^{(\xi\lambda)}(\mathbf{k}') \right\rangle \left\langle G_A^{(\xi\lambda)}(\mathbf{k}') \right\rangle \left| T^{(\xi\lambda)}_{\mathbf{k}'_\parallel \mathbf{k}_\parallel} \right|^2 \boldsymbol{\Gamma}_{RA}(\mathbf{k}', E). \quad (3)$$

The vertex function must be parallel to the $\mathbf{k}$ vector in Eq. (3), such that we write

$$\mathbf{\Gamma}_{RA}(\mathbf{k},E)=\gamma(\mathbf{k},E)\mathbf{k}, \tag{4}$$

and given the expression for the retarded Green's functions in Eq. (7) of the main article, in terms of the relaxation time we have

$$\left\langle G_R^{(\xi\lambda)}(\mathbf{k})\right\rangle=\frac{1}{E-\mathcal{E}_{\mathbf{k}}^{(\xi\lambda)}+\frac{i\,\xi\lambda\hbar}{2\tau^{(\xi\lambda)}(k)}}. \tag{5}$$

Therefore, the product of the retarded and advanced averaged Green's functions is

$$\left\langle G_R^{(\xi\lambda)}(\mathbf{k})\right\rangle\left\langle G_A^{(\xi\lambda)}(\mathbf{k})\right\rangle=\frac{2\pi}{\hbar}\tau^{(\xi\lambda)}(k)\frac{\frac{\hbar}{2\pi\tau^{(\xi\lambda)}(k)}}{\left(E-\mathcal{E}_{\mathbf{k}}^{(\xi\lambda)}\right)^2+\left(\frac{\hbar}{2\tau^{(\xi\lambda)}(k)}\right)^2}. \tag{6}$$

In the limit of low concentration of dislocations, i.e., $n_d\to 0$, is proportional to a very narrow Lorentzian distribution, with support at $E=\mathcal{E}_{\mathbf{k}}^{(\xi\lambda)}$; for calculation purposes can be approximated by a delta function. Then

$$\left\langle G_R^{(\xi\lambda)}(\mathbf{k})\right\rangle\left\langle G_A^{(\xi\lambda)}(\mathbf{k})\right\rangle\to\frac{2\pi\tau^{(\xi\lambda)}(k)}{\hbar}\delta\left(E-\mathcal{E}_{\mathbf{k}}^{(\xi\lambda)}\right). \tag{7}$$

Then, we obtain a secular integral equation for the scalar function $\gamma(\mathbf{k},E)$

$$\gamma(\mathbf{k},E)=1+n_d\frac{2\pi}{\hbar}\int\frac{d^2k'_{\parallel}}{(2\pi)^2}\tau^{(\xi\lambda)}(k')\left|T_{\mathbf{k}'_{\parallel}\mathbf{k}_{\parallel}}^{(\xi\lambda)}\right|^2\delta(E-\xi\lambda\hbar v_F k')\;\gamma(\mathbf{k}',E)\frac{\mathbf{k}\cdot\mathbf{k}'}{k^2}. \tag{8}$$

3 Onsager and transport coefficients

Let us compute the correlation functions

$$L_{\alpha\beta}^{(ij)}=-T\int_0^\infty dt\,e^{-s\,t}\int_0^\beta d\beta'\;\mathrm{Tr}\left[\hat{\rho}_0\hat{j}_{i,\alpha}(-t-i\hbar\beta')\hat{j}_{j,\beta}\right]. \tag{9}$$

We start with Eq. (9) and take the trace in the complete and orthonormal basis $\{|\Psi_{\mathbf{k},\lambda}\rangle\}$ of the total Hamiltonian, such that $\hat{\rho}_0\,|\Psi_{\mathbf{k},\lambda}\rangle=\rho_0\left(\mathcal{E}_{\mathbf{k}}^{(\xi\lambda)}\right)|\Psi_{\mathbf{k},\lambda}\rangle$. Then, using the representation of operators in the Heisenberg picture, and $\rho_0(E)=e^{-\beta(E-\mu)}/\Xi$, we have

$$
\begin{aligned}
L^{(ij;\xi\lambda)}_{\alpha\beta}(\mathbf{r},\mathbf{r}') &= -T\int_0^\infty dt\, e^{-s\,t}\int_0^\beta d\beta' \sum_{\lambda,\lambda'}\int\frac{d^3k}{(2\pi)^3}\int\frac{d^3k'}{(2\pi)^3} \\
&\quad\times \left\langle \Psi_{\mathbf{k},\lambda}\middle|\hat{\rho}_0 e^{i(-t-i\hbar\beta')\hat{H}^\xi/\hbar}\hat{j}_{i,\alpha}(\mathbf{r})e^{i(t+i\hbar\beta')\hat{H}^\xi/\hbar}\middle|\Psi_{\mathbf{k}',\lambda'}\right\rangle\left\langle \Psi_{\mathbf{k}',\lambda'}\middle|\hat{j}_{j,\beta}(\mathbf{r}')\middle|\Psi_{\mathbf{k},\lambda}\right\rangle \\
&= -\hbar T\sum_{\lambda,\lambda'}\int\frac{d^3k}{(2\pi)^3}\int\frac{d^3k'}{(2\pi)^3}\left[\frac{-i\left(\mathcal{E}^{(\xi\lambda)}_{\mathbf{k}}-\mathcal{E}^{(\lambda',\xi)}_{\mathbf{k}'}\right)+\hbar\, s}{\left(\mathcal{E}^{(\xi\lambda)}_{\mathbf{k}}-\mathcal{E}^{(\lambda',\xi)}_{\mathbf{k}'}\right)^2+\hbar^2s^2}\right] \\
&\quad\times\left[\frac{\rho_0\left(\mathcal{E}^{(\xi\lambda)}_{\mathbf{k}}\right)-\rho_0\left(\mathcal{E}^{(\lambda',\xi)}_{\mathbf{k}'}\right)}{\left(\mathcal{E}^{(\xi\lambda)}_{\mathbf{k}}-\mathcal{E}^{(\lambda',\xi)}_{\mathbf{k}'}\right)}\right]\left\langle \Psi_{\mathbf{k},\lambda}\middle|\hat{j}_{i,\alpha}(\mathbf{r})\middle|\Psi_{\mathbf{k}',\lambda'}\right\rangle\left\langle \Psi_{\mathbf{k}',\lambda'}\middle|\hat{j}_{j,\beta}(\mathbf{r}')\middle|\Psi_{\mathbf{k},\lambda}\right\rangle. \qquad (10)
\end{aligned}
$$

The numerator of the first square bracket contains a term, $-i\left(\mathcal{E}^{(\xi\lambda)}_{\mathbf{k}}-\mathcal{E}^{(\lambda',\xi)}_{\mathbf{k}'}\right)$, which is odd with respect to the integration variables. Consequently, this term does not contribute to the overall value. As anticipated, there is no imaginary part remaining. To calculate the real part, we employ

$$
\lim_{s\to 0^+}\frac{\hbar\, s}{\left(\mathcal{E}^{(\xi\lambda)}_{\mathbf{k}}-\mathcal{E}^{(\lambda',\xi)}_{\mathbf{k}'}\right)^2+\hbar^2s^2} = \pi\delta\left(\mathcal{E}^{(\xi\lambda)}_{\mathbf{k}}-\mathcal{E}^{(\lambda',\xi)}_{\mathbf{k}'}\right). \qquad (11)
$$

Now, it is important to notice that the equilibrium average of the statistical operator produces the Fermi distribution function. Then, for the term in the second square bracket we have

$$
\left[\frac{\rho_0\left(\mathcal{E}^{(\xi\lambda)}_{\mathbf{k}}\right)-\rho_0\left(\mathcal{E}^{(\lambda',\xi)}_{\mathbf{k}'}\right)}{\left(\mathcal{E}^{(\xi\lambda)}_{\mathbf{k}}-\mathcal{E}^{(\lambda',\xi)}_{\mathbf{k}'}\right)}\right]\delta\left(\mathcal{E}^{(\xi\lambda)}_{\mathbf{k}}-\mathcal{E}^{(\lambda',\xi)}_{\mathbf{k}'}\right) = \left.\frac{\partial f_0(E)}{\partial E}\right|_{E=\mathcal{E}^{(\xi\lambda)}_{\mathbf{k}}}\delta\left(\mathcal{E}^{(\xi\lambda)}_{\mathbf{k}}-\mathcal{E}^{(\lambda',\xi)}_{\mathbf{k}'}\right). \qquad (12)
$$

Thus, using the general expression for the current operators, i.e., $\hat{\mathbf{j}}^\xi_i(\mathbf{r}) = \xi v_F(\hat{H}^\xi-\mu)^{i-1}\,|\mathbf{r}\rangle\boldsymbol{\sigma}\langle\mathbf{r}|$, and the properties of the spectral function as presented in Ref. [2] we get

$$
L^{(ij;\xi\lambda)}_{\alpha\beta}(\mathbf{r},\mathbf{r}') = \frac{\hbar v_F^2 T}{2\pi}\int_{-\infty}^{\infty}dE\left(-\frac{\partial f_0(E)}{\partial E}\right)(E-\mu)^{i+j-2}\,\mathrm{Tr}\,\sigma_\alpha\boldsymbol{\mathcal{A}}^\xi(\mathbf{r},\mathbf{r}';E)\sigma_\beta\boldsymbol{\mathcal{A}}^\xi(\mathbf{r}',\mathbf{r};E), \qquad (13)
$$

The form of the spectral density in coordinates space is

$$
\boldsymbol{\mathcal{A}}^\xi(\mathbf{r},\mathbf{r}';E) = \int\frac{d^3k}{(2\pi)^3}e^{i\mathbf{k}\cdot(\mathbf{r}-\mathbf{r}')}\sum_\lambda\left(\sigma_0+\lambda\frac{\boldsymbol{\sigma}\cdot\mathbf{k}}{|\mathbf{k}|}\right)\mathcal{A}^{(\xi\lambda)}(k). \qquad (14)
$$

If we insert this form into the Eq.(13), we have

$$L_{\alpha\beta}^{(ij;\xi\lambda)}(\mathbf{r}-\mathbf{r}') = \int \frac{d^3q}{(2\pi)^3} e^{i\mathbf{q}\cdot(\mathbf{r}-\mathbf{r}')} \left(\frac{\hbar v_F^2 T}{2\pi}\right) \int_{-\infty}^{\infty} dE \left(-\frac{\partial f_0(E)}{\partial E}\right) \times (E-\mu)^{i+j-2} \int \frac{d^3k}{(2\pi)^3} \operatorname{Tr} \sigma_\alpha \boldsymbol{\mathcal{A}}^\xi(\mathbf{k}+\mathbf{q};E)\sigma_\beta \boldsymbol{\mathcal{A}}^\xi(\mathbf{k};E) = \int \frac{d^3q}{(2\pi)^3} e^{i\mathbf{q}\cdot(\mathbf{r}-\mathbf{r}')} L_{\alpha\beta}^{(ij)}(\mathbf{q};T). \tag{15}$$

Let us define $\mathbf{R} = \mathbf{r}-\mathbf{r}'$. Finally, we compute the corresponding Onsager coefficient as

$$L_{\alpha\beta}^{(ij;\xi\lambda)}(T) = \int d^3R\, L_{\alpha\beta}^{(ij)}(\mathbf{R}) = \int d^3q \left[\int \frac{d^3R}{(2\pi)^3} e^{i\mathbf{q}\cdot\mathbf{R}}\right] L_{\alpha\beta}^{(ij)}(\mathbf{q};T) = \int d^3q\, \delta^{(3)}(\mathbf{q}) L_{\alpha\beta}^{(ij)}(\mathbf{q};T). \tag{16}$$

Then, the Onsager coefficientes are computed from their Fourier transforms by taking the limit of $\mathbf{q}\to\mathbf{0}$. We have

$$L_{\alpha\beta}^{(ij;\xi\lambda)}(T) = \lim_{\mathbf{q}\to\mathbf{0}} L_{\alpha\beta}^{(ij;\xi\lambda)}(\mathbf{q};T) \tag{17}$$
$$= \frac{\hbar v_F^2 T}{2\pi} \int_{-\infty}^{\infty} dE \left(-\frac{\partial f_0(E)}{\partial E}\right) (E-\mu)^{i+j-2} \times \int \frac{d^3k}{(2\pi)^3} \mathcal{A}^{(\xi\lambda)}(k;E)\mathcal{A}^{(\xi\lambda)}(k;E) \operatorname{Tr}\left[\sigma_\alpha \left(\sigma_0 + \lambda\frac{\boldsymbol{\sigma}\cdot\mathbf{k}}{k}\right)\sigma_\beta \left(\sigma_0 + \lambda\frac{\boldsymbol{\sigma}\cdot\mathbf{k}}{k}\right)\right]$$

From the $SU(2)$ algebra, we can readily obtain the trace

$$\operatorname{Tr}\left[\sigma_\alpha \left(\sigma_0 + \lambda\frac{\boldsymbol{\sigma}\cdot\mathbf{k}}{k}\right)\sigma_\beta \left(\sigma_0 + \lambda\frac{\boldsymbol{\sigma}\cdot\mathbf{k}}{k}\right)\right] = 4\frac{k_\alpha k_\beta}{k^2}, \tag{18}$$

and hence we have

$$L_{\alpha\beta}^{(ij;\xi\lambda)}(T) = 4\left(\frac{\hbar v_F^2 T}{2\pi}\right) \int_{-\infty}^{\infty} dE \left(-\frac{\partial f_0(E)}{\partial E}\right) (E-\mu)^{i+j-2} \int \frac{d^3k}{(2\pi)^3} \frac{k_\alpha k_\beta}{k^2} \mathcal{A}^{(\xi\lambda)}(k;E)\mathcal{A}^{(\xi\lambda)}(k;E). \tag{19}$$

Now, assuming for the moment that the system is homogeneous and isotropic, we can write

$$\int \frac{d^3k}{(2\pi)^3} \frac{k_\alpha k_\beta}{k^2} = \frac{\delta_{\alpha\beta}}{3} \int \frac{d^3k}{(2\pi)^3} \frac{\mathbf{k}\cdot\mathbf{k}}{k^2}. \tag{20}$$

Finally, using the definition of the spectral function in terms of the retarded and advanced disorder-averaged Green's functions,

$$\mathcal{A}^{(\xi\lambda)}(k;E) = i\left[\left\langle G_R^{(\xi\lambda)}(k;E)\right\rangle - \left\langle G_A^{(\xi\lambda)}(k;E)\right\rangle\right], \tag{21}$$

we obtain (in the limit of low concentrations $n_d \ll 1$)

$$L_{\alpha\beta}^{(ij;\xi\lambda)}(T) = \delta_{\alpha\beta}\frac{8}{3}\left(\frac{\hbar v_F^2 T}{2\pi}\right)\int\frac{d^3k}{(2\pi)^3}\int_{-\infty}^{\infty}dE\left(-\frac{\partial f_0(E)}{\partial E}\right)(E-\mu)^{i+j-2}\left\langle G_R^{(\xi\lambda)}(\mathbf{k})\right\rangle\left\langle G_A^{(\xi\lambda)}(\mathbf{k})\right\rangle\frac{\mathbf{k}\cdot\mathbf{k}}{k^2}. \tag{22}$$

Note that the only part that gives a non-zero contribution is the one given by the product of the retarded and the advanced GFs. This is because, as can be seen from Eq.(5), the poles of $\left\langle G_R^{(\xi\lambda)}(\mathbf{k})\right\rangle$ are located entirely in the lower complex half-plane, and therefore, when evaluating the product two $\left\langle G_R^{(\xi\lambda)}(\mathbf{k})\right\rangle$, a contour can be chosen such that the result is zero. The same can be said for the product of two $\left\langle G_A^{(\xi\lambda)}(\mathbf{k})\right\rangle^2$.

Now, by including the vertex corrections, as described in Ref. [2], it formally accounts by replacing one of the factors $\mathbf{k}\to\mathbf{\Gamma}_{RA}(\mathbf{k};E)$ in Eq.(22), such that we have

$$L_{\alpha\beta}^{(ij;\xi\lambda)}(T) = \delta_{\alpha\beta}\frac{8}{3}\left(\frac{\hbar v_F^2 T}{2\pi}\right)\int\frac{d^3k}{(2\pi)^3}\int_{-\infty}^{\infty}dE\left(-\frac{\partial f_0(E)}{\partial E}\right)(E-\mu)^{i+j-2}\left\langle G_R^{(\xi\lambda)}(\mathbf{k})\right\rangle\left\langle G_A^{(\xi\lambda)}(\mathbf{k})\right\rangle\frac{\mathbf{k}\cdot\mathbf{\Gamma}_{RA}(\mathbf{k};E)}{k^2}. \tag{23}$$

From the analysis presented in Section 2, we know that the vertex function $\mathbf{\Gamma}_{RA}(\mathbf{k},E)$ is given as the solution to the *Bethe-Salpeter equation*, where $\mathbf{\Gamma}_{RA}(\mathbf{k},E) = \gamma(\mathbf{k},E)\,\mathbf{k}$ and the scalar function $\gamma(\mathbf{k},E)$ satisfies the secular equation in Eq. (8). It should be noted that the result in Eq. (8) needs to be substituted in Eq.(23). From this, it is evident that at low temperatures, an exact solution is possible since the derivative of the Fermi distribution, given by

$$-\frac{\partial f_0(E)}{\partial E} = \frac{1}{k_B T}f_0(E)\left[1-f_0(E)\right], \tag{24}$$

has a compact support at the Fermi energy. Therefore, we can evaluate $\gamma(k;E)$ and $\tau^{(\xi\lambda)}(k)$ at the Fermi momentum k_F^ξ in Eq.(8) to obtain

$$\gamma(k_F^\xi) = 1 + \gamma(k_F^\xi)\tau^{(\xi\lambda)}(k_F^\xi)\,n_d\frac{2\pi}{\hbar}\int\frac{d^2k'_\parallel}{(2\pi)^2}\left|T_{\mathbf{k'}_\parallel\mathbf{k}_\parallel}^{(\xi\lambda)}\right|^2\delta\left(\mathcal{E}_{\mathbf{k}_F}^{(\xi\lambda)} - \mathcal{E}_{\mathbf{k'}}^{(\xi\lambda)}\right)\frac{\mathbf{k}\cdot\mathbf{k'}}{k^2}. \tag{25}$$

Taking into account that $\cos\phi' = \mathbf{k}\cdot\mathbf{k}'/k^2$ and defining

$$\frac{1}{\tau_1^{(\xi\lambda)}(k_F^\xi)} = n_d \frac{2\pi}{\hbar}\int \frac{d^2k'_\parallel}{(2\pi)^2}\delta\left(\mathcal{E}_{\mathbf{k}_F}^{(\xi\lambda)} - \mathcal{E}_{\mathbf{k}'}^{(\xi\lambda)}\right)\left|T_{\mathbf{k}'_\parallel\mathbf{k}_\parallel}^{(\xi\lambda)}\right|^2 \cos\phi', \tag{26}$$

we can solve for $\gamma(k_F^\xi)$, i.e.,

$$\gamma(k_F^\xi) = \frac{\tau_1^{(\xi\lambda)}(k_F^\xi)}{\tau_1^{(\xi\lambda)}(k_F^\xi) - \tau^{(\xi\lambda)}(k_F^\xi)}. \tag{27}$$

Substituting the result in Eq.(7), the derivative of the Fermi's distribution in Eq.(24), and $\gamma(\mathbf{k})$ from Eq. (27) in the Eq.(23), we get for the bulk Onsager coefficients

$$L_{\alpha\beta}^{(ij;\xi\lambda)}(T) = \delta_{\alpha\beta}\frac{8v_F^2}{3k_B}\gamma(k_F^\xi)\tau^{(\xi\lambda)}(k_F^\xi)\int \frac{d^3k}{(2\pi)^3} f_0\left(\mathcal{E}_{\mathbf{k}}^{(\xi\lambda)}\right)\left[1 - f_0\left(\mathcal{E}_{\mathbf{k}}^{(\xi\lambda)}\right)\right]\left(\mathcal{E}_{\mathbf{k}}^{(\xi\lambda)} - \mu\right)^{i+j-2}. \tag{28}$$

But the energy is of the form $\mathcal{E}_{\mathbf{k}}^{(\xi\lambda)} = \xi\lambda\hbar v_F k$, then it only depends on the magnitude of $\mathbf{k}$ and we can perform the angular integration. Then

$$L_{\alpha\beta}^{(ij;\xi\lambda)}(T) = \delta_{\alpha\beta}\frac{4v_F^2}{3\pi^2 k_B}\tau_{\mathrm{tr}}^{(\xi\lambda)}(k_F^\xi)\int_0^\infty dk\, k^2\, f_0\left(\mathcal{E}_{\mathbf{k}}^{(\xi\lambda)}\right)\left[1 - f_0\left(\mathcal{E}_{\mathbf{k}}^{(\xi\lambda)}\right)\right]\left(\mathcal{E}_{\mathbf{k}}^{(\xi\lambda)} - \mu\right)^{i+j-2}. \tag{29}$$

Here, the total *transport relaxation time* is defined by

$$\begin{aligned}\frac{1}{\tau_{\mathrm{tr}}^{(\xi\lambda)}(k_F^\xi)} &= \frac{1}{\tau^{(\xi\lambda)}(k_F^\xi)} - \frac{1}{\tau_1^{(\xi\lambda)}(k_F^\xi)} \\ &= \frac{2\pi n_d}{\hbar}\int \frac{d^2k'}{(2\pi)^2}\delta(\hbar v_F k_F^\xi - \hbar v_F k')\left|T_{\mathbf{k}'_\parallel\mathbf{k}_\parallel}^{(\xi\lambda)}\right|^2 (1 - \cos\phi').\end{aligned} \tag{30}$$

The closed expression for the transport relaxation time in terms of the scattering phase shifts $\delta_m(k)$ is

$$\frac{1}{\tau_{\mathrm{tr}}^{(\xi\lambda)}(k_F^\xi)} = \frac{2n_d v_F}{k_F^\xi}\sum_{m=-\infty}^{\infty}\sin^2\left[\delta_m(k_F^\xi) - \delta_{m-1}(k_F^\xi)\right], \tag{31}$$

which corresponds to Eq. (31) in the main text. Now, we compute the integral in Eq. (29). First, we note

that $f_0(\varepsilon)\left[1-f_0(\varepsilon)\right]=f_0(\varepsilon)f_0(-\varepsilon)$ is an even function of its argument. Then we can write

$$f_0\left(\mathcal{E}_{\mathbf{k}}^{(\xi\lambda)}\right)\left[1-f_0\left(\mathcal{E}_{\mathbf{k}}^{(\xi\lambda)}\right)\right]=\frac{e^{\xi\lambda\frac{(\hbar v_F k-\xi\lambda\mu)}{k_BT}}}{\left(e^{\xi\lambda\frac{(\hbar v_F k-\xi\lambda\mu)}{k_BT}}+1\right)^2}=\frac{e^{\frac{(\hbar v_F k-\xi\lambda\mu)}{k_BT}}}{\left(e^{\frac{(\hbar v_F k-\xi\lambda\mu)}{k_BT}}+1\right)^2}, \tag{32}$$

because $\xi\lambda=\pm1$ depending on λ and ξ have the same or different sign. We make the change of variables

$$x=\frac{\hbar v_F}{k_BT}k,\quad \tilde{\mu}=\frac{\xi\lambda\mu}{k_BT}. \tag{33}$$

Then,

$$f_0(1-f_0)=\frac{e^{x-\tilde{\mu}}}{(e^{x-\tilde{\mu}}+1)^2}=\frac{\partial}{\partial\tilde{\mu}}\left(\frac{1}{e^{x-\tilde{\mu}}+1}\right). \tag{34}$$

Making this change of variables we have

$$L_{\alpha\beta}^{(ij;\xi\lambda)}(T)=\delta_{\alpha\beta}\frac{4v_F^2}{3\pi^2k_B}\tau_{\rm tr}^{(\xi\lambda)}(k_F^{\xi})\left(\frac{k_BT}{\hbar v_F}\right)^3(\xi\lambda k_BT)^{i+j-2}\int_0^{\infty}dx\frac{x^2(x-\tilde{\mu})^{i+j-2}e^{x-\tilde{\mu}}}{(e^{x-\tilde{\mu}}+1)^2}. \tag{35}$$

This last result can be written in the form

$$L_{\alpha\beta}^{(ij;\xi\lambda)}(T)=\frac{4\delta_{\alpha\beta}}{3\pi^2k_Bv_F}\left(\frac{k_BT}{\hbar}\right)^3\tau_{\rm tr}^{(\xi\lambda)}(k_F^{\xi})\,\tilde{\ell}^{(ij)}, \tag{36}$$

where

$$\tilde{\ell}^{(ij;\xi\lambda)}=(\xi\lambda k_BT)^{i+j-2}\int_0^{\infty}dx\frac{x^2(x-\tilde{\mu})^{i+j-2}e^{x-\tilde{\mu}}}{(e^{x-\tilde{\mu}}+1)^2}. \tag{37}$$

In this form is clear that the integrals of interest are of the form

$$I_n=\frac{d}{d\tilde{\mu}}\int_0^{\infty}\frac{x^n}{e^{x-\tilde{\mu}}+1}dx, \tag{38}$$

for $n=2,3,4$. Using the integral representation of the Polylogarithm function

$$-\mathrm{Li}_s(-z)=\frac{1}{\Gamma(s)}\int_0^{\infty}\frac{x^{s-1}}{e^x/z+1}dx, \tag{39}$$

and the derivative relation

$$\frac{d}{d\tilde{\mu}}\mathrm{Li}_s(-e^{\tilde{\mu}}) = \mathrm{Li}_{s-1}(-e^{\tilde{\mu}}), \tag{40}$$

one can show that the integral of interest is

$$I_n = -n!\;\mathrm{Li}_n(-e^{\tilde{\mu}}). \tag{41}$$

Thus, for $i = j = 1$ we have

$$\tilde{\ell}^{(11;\xi\lambda)} = I_2 = -2\mathrm{Li}_2\left(-e^{\frac{\xi\lambda\hbar v_F k_F^\xi}{k_B T}}\right), \tag{42}$$

but $\Delta\mathcal{E}_F^\xi = \mathcal{E}_F - \mathcal{E}_W^\xi = \xi\lambda\hbar v_F k_F^\xi$ is simply the energy difference between the Fermi level (chemical potential) and the Weyl node. After replacing this in Eq.(36) we get

$$L_{\alpha\beta}^{(11;\xi\lambda)}(T) = -\frac{8\delta_{\alpha\beta}}{3\pi^2 k_B v_{F,\alpha}^{(\xi\lambda)}}\left(\frac{k_B T}{\hbar}\right)^3 \tau_{\mathrm{tr}}^{(\xi\lambda)}(k_F^\xi)\mathrm{Li}_2\left(-e^{\frac{\Delta\mathcal{E}_F^\xi}{k_B T}}\right). \tag{43}$$

For the case $i = 1, j = 2$ we have

$$\begin{aligned}\tilde{\ell}^{(12;\xi\lambda)} &= \tilde{\ell}^{(21;\xi\lambda)} = \xi\lambda k_B T\left[I_3 - \tilde{\mu} I_2\right] \\ &= -2\xi\lambda k_B T\left[3\,\mathrm{Li}_3\left(-e^{\frac{\xi\lambda\hbar v_F k_F^\xi}{k_B T}}\right) - \frac{\xi\lambda\hbar v_F k_F^\xi}{k_B T}\,\mathrm{Li}_2\left(-e^{\frac{\xi\lambda\hbar v_F k_F^\xi}{k_B T}}\right)\right],\end{aligned} \tag{44}$$

and the corresponding Onsager coefficient is obtained replacing this result in Eq.(36)

$$L_{\alpha\beta}^{(12;\xi\lambda)}(T) = L_{\alpha\beta}^{(21;\xi\lambda)}(T) = -\frac{8\xi\lambda\hbar\delta_{\alpha\beta}}{3\pi^2 k_B v_{F,\alpha}^{(\xi\lambda)}}\left(\frac{k_B T}{\hbar}\right)^4 \tau_{\mathrm{tr}}^{(\xi\lambda)}(k_F^\xi)\left[3\,\mathrm{Li}_3\left(-e^{\frac{\Delta\mathcal{E}_F^\xi}{k_B T}}\right) - \frac{\Delta\mathcal{E}_F^\xi}{k_B T}\,\mathrm{Li}_2\left(-e^{\frac{\Delta\mathcal{E}_F^\xi}{k_B T}}\right)\right], \tag{45}$$

Finally, for the case $i = j = 2$, we get

$$\begin{aligned}\tilde{\ell}^{(22;\xi\lambda)} &= (k_B T)^2\left[I_4 - 2\tilde{\mu}\,I_3 + \tilde{\mu}^2\,I_2\right] \\ &= -2(k_B T)^2\left[12\,\mathrm{Li}_4\left(-e^{\frac{\xi\lambda\hbar v_F k_F^\xi}{k_B T}}\right) - 6\xi\lambda\frac{\hbar v_F k_F^\xi}{k_B T}\,\mathrm{Li}_3\left(-e^{\frac{\xi\lambda\hbar v_F k_F^\xi}{k_B T}}\right) + \left(\frac{\hbar v_F k_F^\xi}{k_B T}\right)^2\mathrm{Li}_2\left(-e^{\frac{\xi\lambda\hbar v_F k_F^\xi}{k_B T}}\right)\right].\end{aligned} \tag{46}$$

Replacing this last result in Eq.(36) we obtain

$$L_{\alpha\beta}^{(22;\xi\lambda)}(T) = -\frac{8\hbar^2\delta_{\alpha\beta}}{3\pi^2 k_B v_{F,\alpha}^{(\xi\lambda)}}\left(\frac{k_B T}{\hbar}\right)^5\left[12\,\mathrm{Li}_4\left(-e^{\frac{\Delta\varepsilon_F^\xi}{k_B T}}\right) - \frac{6\,\Delta\mathcal{E}_F^\xi}{k_B T}\,\mathrm{Li}_3\left(-e^{\frac{\Delta\varepsilon_F^\xi}{k_B T}}\right) + \left(\frac{\Delta\mathcal{E}_F^\xi}{k_B T}\right)^2\mathrm{Li}_2\left(-e^{\frac{\Delta\varepsilon_F^\xi}{k_B T}}\right)\right], \tag{47}$$

References

[1] Daniel Bonilla, Enrique Muñoz, and Rodrigo Soto-Garrido. Thermo-magneto-electric transport through a torsion dislocation in a type i weyl semimetal. *Nanomaterials*, 11(11):2972, 2021. ISSN 2079-4991. doi: 10.3390/nano11112972. URL `https://www.mdpi.com/2079-4991/11/11/2972`.

[2] Daniel Bonilla and Enrique Muñoz. Electronic transport in weyl semimetals with a uniform concentration of torsional dislocations. *Nanomaterials*, 12(20):3711, 2022. ISSN 2079-4991. doi: 10.3390/nano12203711. URL `https://www.mdpi.com/2079-4991/12/20/3711`.

[3] Davide Grassano, Olivia Pulci, Adriano Mosca Conte, and Friedhelm Bechstedt. Validity of weyl fermion picture for transition metals monopnictides taas, tap, nbas, and nbp from ab initio studies. *Scientific Reports*, 8(1):3534, Feb 2018. ISSN 2045-2322. doi: 10.1038/s41598-018-21465-z. URL `https://doi.org/10.1038/s41598-018-21465-z`.

[4] Chi-Cheng Lee, Su-Yang Xu, Shin-Ming Huang, Daniel S. Sanchez, Ilya Belopolski, Guoqing Chang, Guang Bian, Nasser Alidoust, Hao Zheng, Madhab Neupane, Baokai Wang, Arun Bansil, M. Zahid Hasan, and Hsin Lin. Fermi surface interconnectivity and topology in weyl fermion semimetals taas, tap, nbas, and nbp. *Phys. Rev. B*, 92:235104, Dec 2015. doi: 10.1103/PhysRevB.92.235104. URL `https://link.aps.org/doi/10.1103/PhysRevB.92.235104`.

Tab. 1: Values of the Fermi velocities $v_{F,\alpha}^{(\xi\lambda)}$ in units of 10^5 m/s, reported in literature. In the valence band ($\lambda = -1$) it corresponds to hole velocities. We use the average of the two reported values in each case.

Material	$v_{F,x}^{(++)}$ [3]	$v_{F,x}^{(++)}$ [4]	$v_{F,x}^{(++)}$ av.	$v_{F,x}^{(+-)}$ [3]	$v_{F,x}^{(+-)}$ [4]	$v_{F,x}^{(+-)}$ av.	$v_{F,x}^{(-+)}$ [3]	$v_{F,x}^{(-+)}$ [4]	$v_{F,x}^{(-+)}$ av.	$v_{F,x}^{(--)}$ [3]	$v_{F,x}^{(--)}$ [4]	$v_{F,x}^{(--)}$ av.
TaAs	3.2	2.5	2.85	-5.3	-5.2	- 5.25	2.6	2.4	2.5	-4.3	-4.3	-4.3
TaP	3.7	3.1	3.4	-5.4	-5.7	-5.55	2.0	2.3	2.15	-3.9	-4.1	-4.0
NbAs	3.0	2.5	2.75	-4.8	-4.8	-4.8	2.5	2.4	2.45	-3.2	-3.3	-3.25
NbP	3.0	3.7	3.35	-5.1	-5.7	-5.4	1.7	2.1	1.9	-2.4	-3.2	-2.8
Material	$v_{F,y}^{(++)}$ [3]	$v_{F,y}^{(++)}$ [4]	$v_{F,y}^{(++)}$ av.	$v_{F,y}^{(+-)}$ [3]	$v_{F,y}^{(+-)}$ [4]	$v_{F,y}^{(+-)}$ av.	$v_{F,y}^{(-+)}$ [3]	$v_{F,y}^{(-+)}$ [4]	$v_{F,y}^{(-+)}$ av.	$v_{F,y}^{(--)}$ [3]	$v_{F,y}^{(--)}$ [4]	$v_{F,y}^{(--)}$ av.
TaAs	3.2	1.2	2.2	-1.4	-3.2	-2.3	3.5	3.5	3.5	-1.8	-1.7	-1.75
TaP	3.6	1.5	2.55	-1.5	-3.6	-2.55	3.1	3.0	3.05	-2.1	-2.0	-2.05
NbAs	2.1	1.2	1.65	-1.4	-2.0	-1.7	2.3	2.3	2.3	-1.3	-1.2	-1.25
NbP	2.9	1.5	2.2	-1.6	-3.0	-2.3	2.0	2.1	2.05	-1.7	-1.6	-1.65
Material	$v_{F,z}^{(++)}$ [3]	$v_{F,z}^{(++)}$ [4]	$v_{F,z}^{(++)}$ av.	$v_{F,z}^{(+-)}$ [3]	$v_{F,z}^{(+-)}$ [4]	$v_{F,z}^{(+-)}$ av.	$v_{F,z}^{(-+)}$ [3]	$v_{F,z}^{(-+)}$ [4]	$v_{F,z}^{(-+)}$ av.	$v_{F,z}^{(--)}$ [3]	$v_{F,z}^{(--)}$ [4]	$v_{F,z}^{(--)}$ av.
TaAs	0.2	0.2	0.2	-0.2	-0.2	-0.2	4.4	4.3	4.35	-1.6	-1.6	-1.6
TaP	0.2	0.2	0.2	-0.2	-0.2	-0.2	4.2	4.4	4.3	-1.4	-1.5	-1.45
NbAs	0.1	0.1	0.1	-0.1	-0.1	-0.1	3.6	3.7	3.65	-1.2	-1.1	-1.15
NbP	0.0(3)	0.0(3)	0.0(3)	-0.0(3)	-0.0(3)	-0.0(3)	4.2	3.8	4.0	-1.4	-1.0	-1.2
